# ApicoAP: The First Computational Model for Identifying Apicoplast-Targeted Proteins in Multiple Species of Apicomplexa

**DOI:** 10.1371/journal.pone.0036598

**Published:** 2012-05-04

**Authors:** Gokcen Cilingir, Shira L. Broschat, Audrey O. T. Lau

**Affiliations:** 1 School of Electrical Engineering and Computer Science, Washington State University, Pullman, Washington, United States of America; 2 Department of Veterinary Microbiology and Pathology, Paul G. Allen School for Global Animal Health, Washington State University, Pullman, Washington, United States of America; Institut national de la santé et de la recherche médicale – Institut Cochin, France

## Abstract

**Background:**

Most of the parasites of the phylum Apicomplexa contain a relict prokaryotic-derived plastid called the apicoplast. This organelle is important not only for the survival of the parasite, but its unique properties make it an ideal drug target. The majority of apicoplast-associated proteins are nuclear encoded and targeted post-translationally to the organellar lumen via a bipartite signaling mechanism that requires an N-terminal signal and transit peptide (TP). Attempts to define a consensus motif that universally identifies apicoplast TPs have failed.

**Methodology/Principal Findings:**

In this study, we propose a generalized rule-based classification model to identify apicoplast-targeted proteins (ApicoTPs) that use a bipartite signaling mechanism. Given a training set specific to an organism, this model, called ApicoAP, incorporates a procedure based on a genetic algorithm to tailor a discriminating rule that exploits the known characteristics of ApicoTPs. Performance of ApicoAP is evaluated for four labeled datasets of *Plasmodium falciparum*, *Plasmodium yoelii*, *Babesia bovis*, and *Toxoplasma gondii* proteins. ApicoAP improves the classification accuracy of the published dataset for *P. falciparum* to 94%, originally 90% using PlasmoAP.

**Conclusions/Significance:**

We present a parametric model for ApicoTPs and a procedure to optimize the model parameters for a given training set. A major asset of this model is that it is customizable to different parasite genomes. The ApicoAP prediction software is available at http://code.google.com/p/apicoap/ and http://bcb.eecs.wsu.edu.

## Introduction

The apicoplast is a relict plastid that resides in most of the parasites of the phylum Apicomplexa [1], [2]. Members of this phylum include *Plasmodium falciparum*, the causative agent of the most deadly form of malaria, *Plasmodium yoelii*, another malaria-causing agent, and *Toxoplasma gondii* and *Babesia bovis*, which cause toxoplasmosis and babesiosis, respectively. The apicoplast is an essential organelle for the survival of these parasites [3], [4]. Moreover, many apicoplast proteins and pathways have prokaryotic characteristics due to the organelle's ancestral relationship to bacteria [1], [5]. Because these proteins and pathways are either absent or divergent from those of its eukaryotic host, they are seen as promising drug targets with minimum side effects to the infected host [6], [5]. Most apicoplast proteins are nuclear-encoded and targeted post-translationally to the organellar lumen [7]–[10]. Understanding the metabolic activities performed in the apicoplast is essential for drug target identification, and this requires the ability to detect apicoplast targeting signals in proteins.

Protein import into the lumen of the apicoplast is facilitated by a bipartite signaling mechanism that requires an N-terminal signal peptide (SP) followed by a transit peptide (TP) [9]. Although other mechanisms may exist [11], the bipartite signaling mechanism is most easily recognized. Well-established prediction algorithms exist for determining the existence of an SP in a protein sequence independent of the organism to which it belongs [12]–[15]. In contrast, there is no established computational method that determines the existence of a TP in multiple organisms. In fact, attempts to define a consensus motif that universally identifies apicoplast TPs have failed because preferred amino acids in TP regions are heavily influenced by the Adenine-Thymidine (AT) codon bias of parasitic genomes [16]. For example, the genome of *P. falciparum* is approximately 80% AT-enriched [16], and apicoplast TPs are dominated by amino acids such as asparagine (N) and lysine (K), which exclusively utilize codons lacking Guanine and Cytosine. PlasmoAP, a rule-based prediction method, makes use of this bias and suggests that the anticipated TP region (defined as the region that starts after the predicted SP-cleavage site with a cutoff of 80 amino acids) of apicoplast-targeted proteins (ApicoTPs) must contain an NK-enriched sub-region with a basic to acidic amino acid ratio of at least 5 to 3 [17]. Application of this method to other Apicomplexa with more balanced AT content is not considered reliable. As a result, application of PlasmoAP to the *Babesia bovis* genome revealed only a handful of candidate ApicoTPs in comparison to>460 predicted ApicoTPs in *P. falciparum* [18]. With the sequence completion of several Apicomplexan genomes, there is a pressing need to have a computational method for detecting ApicoTPs that is applicable to different organisms rather than to a single model organism.

PATS [19] and PlasmoAP [17] are the only computational methods described in the literature that detect TP regions in protein sequences. These two methods are specifically designed for the *P. falciparum* proteome. PATS follows a black-box approach that is based on training a neural network over amino acid content-based features harvested from the anticipated TP region (defined as the region that starts after the predicted SP-cleavage site with a cutoff of 78 amino acids). Unlike PlasmoAP, PATS offers predictions only, without providing any understanding of the actual prediction mechanism. As a rule-based method, PlasmoAP holds an advantage over PATS in the sense that it offers insight into the underlying targeting mechanism and allows the formulation of testable hypotheses.

In this paper, we propose a generalized rule-based classification model to identify ApicoTPs that use a bipartite signaling mechanism. Based only on the known characteristics of ApicoTPs, a parametric model is constructed. Given a training set specific to an organism, our model, ApicoAP for *APICO*mplexan *A*picoplast *P*roteins, employs a procedure based on a genetic algorithm to tailor a discriminating rule that maximizes the prediction and generalization performance for the given set. An advantage of ApicoAP is that it is customizable to different organisms when training data are available.

## Materials and Methods

### Selection of a classification model

From a computational point of view, the prediction of a given protein as an ApicoTP or non-ApicoTP can be stated as a binary classification problem, for which we choose ApicoTP as the positive class. It is worth noting that we define the ApicoTP class such that proteins localizing to multiple organelles including the apicoplast are members of this class in addition to proteins localizing only to the apicoplast. In a typical supervised learning setting, a training set containing positive and negative labeled instances is used to learn a mapping from the input to the output. In our case, the goal is to learn a mapping from protein sequences to the binary class labels: ApicoTP and non-ApicoTP. Our machine learning approach towards this goal is to assume a parametric model to define this mapping and estimate model parameters using a training set such that the error for parameter estimates is minimized. This estimation process is often called training. As a result of training, a model with specific parameters, in other words a classifier, is achieved, which can then be employed to predict the labels for new instances [20].

After some consideration, we chose a rule-based approach, similar to the one used by the developers of PlasmoAP [17], as the basis for our classification model. Properties of ApicoTPs were used to construct a generalized rule defined by a set of parameters. After completion of training by means of a genetic algorithm, the resulting classifier was then used to predict a protein sequence as ApicoTP or non-ApicoTP. Before explaining the details of our generalized rule definition, we will discuss the known properties of ApicoTPs that underlie our model.

#### Properties of apicoplast-targeted proteins (ApicoTPs)

A typical nuclear-encoded ApicoTP contains an N-terminal signal peptide (SP) region followed by a transit peptide (TP) region and a mature protein. The SP is removed during co-translational import into the endoplasmic reticulum (ER) and the TP, which guides the protein into the apicoplast, is removed from the mature protein inside the lumen of the apicoplast [9], [21].

Apicoplast TPs vary greatly in length and are biased towards polar (positive charge preferred), basic, and hydrophilic amino acids [17], [22]. A recent study conducted by [23] indicates that TPs are functionally disordered and therefore biased towards amino acids with low helical propensity as well. In addition, it has been shown that the absence of negative charge, in other words the depletion of acidic residues, is important for transit peptide fidelity [17], [22].

Length variance among TP regions of known ApicoTPs points to the possibility that a smaller sub-region of a perhaps larger TP is used by the apicoplast for recognition. This smaller sub-region (hereafter referred to as the pattern *p*) can be expected to embody the aforementioned properties of TP regions. PlasmoAP makes use of this idea by searching for a stretch of 40 amino acids in the anticipated TP region (with a cutoff of 80 amino acids) that is enriched and depleted by certain amino acid groups. Selection of these amino acid groups and cutoff values was performed only for the model organism, *P. falciparum*, which is the main limitation of PlasmoAP for other organisms.

#### Generalized model for apicoplast-targeted proteins (ApicoTPs)

**Figure 1:**
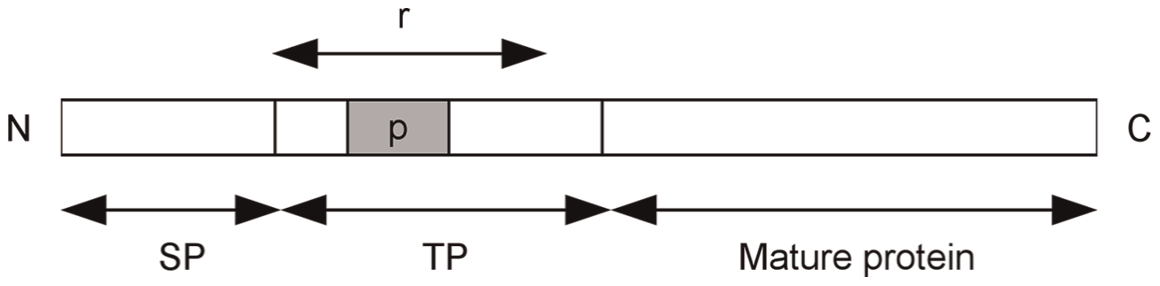
Schematic representation of a typical apicoplast-targeted protein (ApicoTP). A typical ApicoTP with defined regions *r* and *p* is shown, where *r* is the anticipated TP region that starts immediately after the predicted SP cleavage site and *p* is the pattern that contains the core information for predicting an ApicoTP. The pattern *p* is simply a contiguous sub-region of region *r*.

A schematic representation of a typical ApicoTP is given in Figure 1. Because the TP region can be variable in length and in most cases its exact length is unknown, the region *r* is introduced, which represents the anticipated TP region. The region *r* starts immediately after the predicted SP cleavage site and has a length of at most *L_r_*. A pattern *p* with length *L_p_* is assumed to exist in region *r*, which contains the core information that indicates whether the protein under consideration is an ApicoTP. The pattern *p* is simply a contiguous sub-region of region *r* enriched by amino acids that have low helical propensity or are polar (positive charge preferred), basic, or hydrophilic and depleted of acidic and negative amino acids. {H, K, R} are the amino acids that are polar-positive, basic, and highly hydrophilic. {N, Q} are the amino acids that are polar-neutral and highly hydrophilic. {S, P, Y} are moderately hydrophilic amino acids that have low helical propensity. We refer to these eight amino acids as the *preferred residue set (PRS)*. {E, D} are the amino acids that are polar-negative and acidic with high helical propensity. We refer to these as the *avoided residue set (ARS)*. We determined these sets using Chou-Fasman [24] helical propensity predictions and the Kyte-Doolittle [25] hydropathy index.

The *preferred residue set score (PRSS)* and *avoided residue set score (ARSS)* quantify the existence of *PRS* and *ARS* elements in an arbitrary region *s*. Equations (1) and (2) give the functional forms of these quantities, where *f (x,s)* is the frequency of an amino acid residue *x* in the region *s*. The *PRSS* and *ARSS* are simply the weighted sums of these frequencies. The weight sets **w_1_** and **w_2_** determine the relative influence of the residues in the scoring functions. When a weight is 0, the frequency of the corresponding residue will have no effect on the score, and when it is 1, it will have the maximum effect.
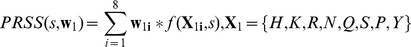
(1)

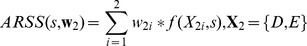
(2)


As stated earlier, the anticipated TP region *r* is assumed to contain a contiguous sub-region *p* with length *L_p_* that embodies the core information for identifying an ApicoTP. We refer to the set containing all contiguous sub-regions with length *L_p_* in *r* as *S_p_*. In an ApicoTP, *p* should have a high *PRSS* and a relatively low *ARSS*. Assuming a linear relationship between the *PRSS* and *ARSS*, the *p-criterion* function given by Eq. (3) defines the criterion for selecting *p* from *S_p_*. Essentially the sub-region with the highest ratio of preferred residues to avoided residues is the optimum choice.
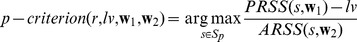
(3)


The limiting value *lv* is an estimate of the *PRSS* when *e* percent of the residues in a region *s* of length *L_s_* are from the *preferred residue set (PRS)*. The reason for including this limiting value is to ensure that a minimum number of elements from the *PRS* are present in the sub-region *p*. Sole absence of avoided residues is insufficient for a protein to be an ApicoTP; a minimum number of preferred residues are required as well. Equation (4) gives the functional form of *lv*.
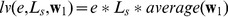
(4)


#### A rule-based classification model for ApicoTPs

The generalized model for ApicoTPs discussed above defines a mapping from protein sequences to *p-criterion* values. In order to use this model as a classifier, a threshold value over *p-criterion* values that separates ApicoTPs from non-ApicoTPs must be determined. This is accomplished via feedback from the training set. We examine possible locations for the threshold and select the one that maximizes the prediction performance of the resulting classifier for the training set. The possible locations for the threshold are the midpoints of each adjacent pair of *p-criterion* values in sorted order. The resulting rule-based classifier classifies a protein sequence with a *p-criterion* value exceeding or equal to the threshold as an ApicoTP.

#### Geometric interpretation of the classification model for ApicoTPs

The *PRSS* and *ARSS*, given by Eqs. (1) and (2), respectively, associated with the sub-region *p* for a given protein sequence map the sequence to a plane in which a discriminating line separates ApicoTPs and non-ApicoTPs. Protein sequences are mapped to a point in the *PRSS*-*ARSS* plane where the ones appearing on or above the discriminating line are predicted to be ApicoTPs. The limiting value *lv*, given by Eq. (4), determines the *PRSS*-intercept of the discriminating line. The threshold over *p-criterion* values, which is determined via feedback from the training set, gives the slope of this line.

If the *ARSS* is zero and the *PRSS* is greater than or equal to the limiting value *lv*, a sequence should be mapped to the ApicoTP region of the *PRSS*-*ARSS* plane, but the *p-criterion* value is undefined because the denominator in Eq. (3) is zero. For such cases, we set the *p-criterion* to be sufficiently large to ensure mapping of the sequence into the ApicoTP region. When the *PRSS* is smaller than *lv* and the *ARSS* is zero, the *p-criterion* is set sufficiently low to ensure mapping of the sequence into the non-ApicoTP region below the discriminating line.

The parameters for the rule-based classification model used in ApicoAP, including the weights, *L_p_*, *L_r_*, and *e*, are optimized using a genetic algorithm as described below, but before discussing our optimization method we discuss another requirement for identifying an ApicoTP with a bipartite signaling mechanism, the presence of a signal peptide.

#### Signal peptide identification

Implicit in our generalized model is that an ApicoTP contains an SP because the anticipated TP region *r* starts from the predicted SP cleavage site. We used SignalP 3.0 [26] for SP cleavage site prediction, as it is the tool commonly reported in the literature for Apicomplexan genomes. We considered using the most recent version of this tool, SignalP 4.0 [12], which is believed to perform better at discriminating SP regions from transmembrane domains existing downstream from the N terminus of a sequence. However, we observed that SignalP 4.0 predicts significantly fewer SPs than SignalP 3.0 for Apicomplexan genomes. For example, according to SignalP 3.0 the *P. falciparum* genome contains about 1100 SPs, but SignalP 4.0 identifies only about 600 SPs. Neither of these tools is trained or tested on Apicomplexan genomes because no Apicomplexan protein has been experimentally confirmed to contain an SP. Further study is needed on Apicomplexan genomes to assess the possible causes for the difference in the number of predictions.

### Optimizing model parameters

A prediction performance measure calculated with a given labeled dataset demonstrates how well the classification model performs on the available data, but it does not predict how well a classifier can be expected to perform in practice. Instead, for our optimization criterion we use the *expected* prediction performance of a model, i.e., how well it is expected to generalize to new data instances; this can be estimated using a cross-validation procedure. In n-fold cross validation, a given dataset is randomly divided into n subsets of equal size. A classifier is trained n times by setting aside one distinct set for validation and using the remaining n-1 sets for training. The average prediction performance for the validation sets gives an estimate of the expected prediction performance of the classifier [20].

We use Matthews Correlation Coefficient (MCC) as our performance measure; the MCC is known as a balanced measure because it weights a true positive prediction and a true negative prediction equally regardless of how imbalanced a test set might be [27]. The more commonly used performance measure, accuracy, is biased toward classifiers that tend to do better on the majority class. The rule-based classification model used in ApicoAP requires several parameters: the weights that are used to calculate the *PRSS* and *ARSS*, the region length *L_r_*, the pattern length *L_p_*, and the limiting percentage *e* from which the limiting value *lv* is determined. An optimization procedure based on a genetic algorithm is applied to determine the set of parameters that produces the model with the maximum expected prediction performance. The problem of choosing the best classification model parameters among all possibilities is characterized as a search problem in which the parameter space is examined using the expected prediction performance as the objective function, calculated using the MCC measure.

#### A brief overview of genetic algorithms

A genetic algorithm (GA) is a heuristic search method inspired by Darwinian evolution [28]. Based on the principle of “survival of the fittest,” a GA maintains a set of candidate solutions called individuals, represented by a set of genes, and applies combination and transformation operations on individuals analogous to crossover and mutation operations in actual genes. A typical iteration for a GA involves selection of the fittest individuals (solutions with highest objective function values), application of the crossover operation to these individuals, generation of random mutations within the newly produced individuals (offspring), and replacement of a percentage of the total population by these offspring. This simulation of evolution on solution instances undergoes several iterations until the stop condition is reached. At this point, the algorithm returns the optimal solution achieved via the iterations.

The power of genetic algorithms comes from the employment of fitness-based selection and genetic operators (crossover and mutation) during reproduction [29]. Fitness-based selection of individuals for reproduction enables the fittest ones to have offspring via the crossover operator, which enables the exchange of genetic information between parents. If we assume that each individual ideally captures different features of the global optima, combining subparts of these individuals from multiple parents on a single offspring greatly speeds up the process of reaching optima. This phenomenon is known as *implicit parallelism* in a GA [30], [31]. The mutation operator introduces localized changes in offspring, which is essential for sustaining exploration in the search space. Mutations introduce the genetic diversity that is not necessarily represented in a population but that may be needed to reach a global optimum.

Many variations of GAs exist in the literature. One can maintain a single population or multiple populations in parallel. If multiple populations are evolved in parallel, migration among them during each iteration can be allowed either for the fittest or for random individuals. At each iteration, the next population may or may not overlap with the previous one.

#### The genetic algorithm for ApicoAP

In the genetic algorithm used in ApicoAP, an individual is represented by a real-valued parameter set containing ten weights, one region length *L_r_*, one pattern length *L_p_*, and one limiting percentage parameter *e*. To simplify the problem, we introduced constraints on the possible values of each parameter. Weight values can be 0, 0.5, or 1. Region length values can be between 60 and 90 with increments of 5. Pattern length values can be between 15 and 40 with increments of 1. Limiting percentage values can be between 0.2 and 0.4 with increments of 0.05. All ranges were determined by experimentation with the training portion of the available data. Experiments conducted with longer region and pattern lengths did not result in significant differences in the rules or performance indicating that the lengths chosen are sufficient.

Uniform crossover and point mutation were defined, and the initial crossover and mutation probabilities were chosen to be 1.0 and 0.1, respectively. Four parallel populations containing 40 individuals were used, and migration was allowed (at each iteration) for the two fittest individuals. Populations were set to be overlapping where 15 individuals were replaced by the newly generated offspring at all iterations. A large number of populations with many individuals are desirable, but efficiency in the computational time required for optimization is also a concern. The replacement percentage and migration limit often determine how quickly population diversities converge to zero, but reaching this state too quickly is undesirable because a local optimum rather than a global optimum is likely to be reached. Maintenance of diverse populations is important for increasing the likelihood of reaching the global optimum of the search space. Thus, in determining parameters there is a tradeoff between time efficiency and maintenance of diverse populations.

To avoid local optimum traps, we implemented a mechanism to monitor population diversities and took preventive action when needed by gradually increasing the mutation rate and by changing the crossover selection criterion from fittest to random. When 30 generations had passed without achieving an improvement in the optimal solution, we stopped the search. Although additional mechanisms were implemented to avoid local optimum traps, several runs were performed to insure an optimal solution had been reached.

### Datasets

To evaluate the performance of ApicoAP, we used five labeled sets of protein sequences from *P. falciparum*, *P. yoelii*, *B. bovis*, and *T. gondii*, each containing sequences of a single organism. We used the published dataset employed in the development of PlasmoAP [17] for the sole purpose of comparing our method with theirs. In addition, we gathered a new training set for *P. falciparum* proteins that incorporates recent experimental findings. We also gathered novel training sets for *P. yoelii*, *B. bovis*, and *T. gondii*. ApiLoc was used as the main resource for locating experimentally confirmed Apicomplexan proteins.

We obtained experimentally-confirmed ApicoTP proteins from the ApiLoc database (version 3, http://apiloc.bio21.unimelb.edu.au) and identified orthologs of these proteins from the OrthoMCL database (version 5) [32]. Proteins verified as having SPs by SignalP 3.0 were used in our positive training sets. Additional proteins were added to our training sets from references [17], [33]–[38]. Because of the scarcity of experimentally-confirmed *P. yoelii* and *B. bovis* ApicoTPs (only three proteins are confirmed to be ApicoTPs for each organism), we used homology transfer to establish reasonably sized training sets. CDART (Conserved Domain Architecture Retrieval Tool) [39] was employed to infer protein homology relationships by means of domain architecture similarity. See Tables S1, S2, S3, S4 for detailed information on the positive training sets.

We obtained proteins tagged as non-Apicoplast from the ApiLoc database and found orthologs using the OrthoMCL database. The proteins predicted to have an SP region were used in our negative training sets. We also found proteins confirmed to localize to locations other than the apicoplast from the ApiLoc database. We manually eliminated proteins whose confirmed localization does not necessarily rule out apicoplast targeting. For example, we eliminated proteins confirmed to localize to mitochondria, food vacuoles, and the cytoplasm, as dual localization incidents have been reported in the literature involving apicoplasts and these locations. Because very few *P. yoelii* and *B. bovis* non-ApicoTPs have been experimentally confirmed, we added proteins annotated as “variant erythrocyte surface antigen,” “merozoite surface antigen,” and “rhoptry related/associated” to the negative training sets to increase their size. See Tables S5, S6, S7, S8 for detailed information on the negative training sets.

All protein sequences were obtained from EuPathDB (version 2.13) [40], which is the main biological sequence repository for eukaryotic pathogens such as Apicomplexans. Table 1 shows the breakdown of each training set by positive (putative ApicoTPs) and negative (non-ApicoTPs) classes.

**Table 1:** Breakdown of the labeled datasets into positive (ApicoTP) and negative (non-ApicoTP) classes.

Dataset	Number of putative ApicoTPs	Number of putative non-ApicoTPs
P. falciparum*	78	27
*P. falciparum*	47	41
*B. bovis*	28	29
*T. gondii*	35	33
*P. yoelii*	34	36

P. falciparum* refers to the published dataset used in the development of PlasmoAP. We used only the SP-containing portion of this set.

For ApicoAP, only proteins containing an SP were used for training. The published dataset of proteins for *P. falciparum* contains 102 non-ApicoTPs of which 75 lack SPs. As with ApicoAP, PlasmoAP requires a protein to contain an SP for prediction as an ApicoTP. Thus, exclusion of the 75 non-ApicoTPs will not affect comparison of the two methods. In fact, it is likely that a negative training set that includes proteins without SPs may well overstate the actual performance of a classifier given that the objective of such classifiers is to discriminate ApicoTPs from non-ApicoTPs when an SP is present.

## Results

### Evaluation of ApicoAP

ApicoAP was used with the five datasets described in the previous section. To estimate the expected prediction performance of ApicoAP, 3×5 cross validation was employed. A rule-based classifier is trained on a subset of a labeled dataset, which will be referred to as the training-validation set. As discussed earlier, this subset is further divided into training and validation sets, using 3×5 cross validation, to facilitate calculation of the objective function value during the parameter optimization phase. The parameters for our rule-based classifier are optimized in this phase, and the resulting classifier is applied to the remaining set (test set) to assess the performance of the model for unknown data. Fifteen test set samples were used to assess the model performance. The expected prediction performance of ApicoAP was calculated using Matthews Correlation Coefficient (MCC) by averaging the classifier MCCs over these samples.

During parameter optimization, often the parameter set found with the optimum objective value is not unique. Small perturbations of one or more parameters result in different parameter sets with the same optimum objective value. The trained classifiers with these parameter sets sometimes possess different expected prediction performances. In Table 2 we report the averages of minimum, maximum, and average accuracies observed together with the standard deviations. These reflect the worst-case, best-case, and the most-likely expected prediction performances, respectively.

**Table 2:** Averaged expected prediction performance of ApicoAP (standard deviation (sd) in parentheses) for the labeled datasets.

Dataset	Average accuracy (sd)	Minimum accuracy (sd)	Maximum accuracy (sd)
P. falciparum*	0.88 (0.08)	0.87 (0.09)	0.90 (0.07)
*P. falciparum*	0.87 (0.06)	0.84 (0.08)	0.91 (0.05)
*B. bovis*	0.82 (0.06)	0.76 (0.11)	0.87 (0.06)
*T. gondii*	0.83 (0.10)	0.8 (0.11)	0.86 (0.09)
*P. yoelii*	0.85 (0.07)	0.82 (0.09)	0.87 (0.06)

The final classifier for each dataset uses a single parameter set. To form this parameter set we took the averages of the individual parameters obtained during the cross validation procedure. We then adjusted the threshold value taking into consideration the entire labeled dataset. Note that the performance measure used for threshold determination was also the MCC. The resulting classifiers for the four organisms were implemented in the ApicoAP software used for predicting putative ApicoTPs (discussed in detail in the next section). Table 3 lists the performance of ApicoAP for the different classifiers. In contrast to the values given in Table 2, the values in Table 3 do not estimate how well ApicoAP will perform for unknown data but rather how well it performs for the available, labeled data.

**Table 3:** ApicoAP classifier performance on the labeled datasets.

Dataset	True positive count (rate)	True negative count (rate)	Overall accuracy
P. falciparum*	73 (0.94)	26 (0.96)	0.94
*P. falciparum*	46 (0.98)	37 (0.9)	0.94
*B. bovis*	27 (0.96)	26 (0.9)	0.93
*T. gondii*	32 (0.91)	27 (0.82)	0.87
*P. yoelii*	32 (0.94)	33 (0.92)	0.93

A comparison between ApicoAP and PlasmoAP for the published *P. falciparum* dataset is given in Table 4. The values in Table 4 show that ApicoAP provides some improvement in both the true positive rate and the true negative rate, the latter implying fewer false positive predictions.

**Table 4:** Comparison of ApicoAP and PlasmoAP for *P. falciparum* dataset of 78 positives and 27 negatives.

Classifier	True positive count (rate)	True negative count (rate)	Overall accuracy
ApicoAP	73 (0.94)	26 (0.96)	0.94
PlasmoAP	72 (0.92)	22 (0.81)	0.9

### ApicoAP predictions

After a given training set is used in the classification model, a rule-based classifier is obtained that predicts an ApicoTP when the following criteria are met:

- The protein sequence is predicted to contain an SP.
- The region of *L_r_* amino acids following the SP cleavage site contains a pattern of *L_p_* amino acids with a *p-criterion* value greater than or equal to the determined threshold.

The classifiers obtained using the training data available for *P. falciparum*, *P. yoelii*, *B. bovis*, and *T. gondii* are available in the ApicoAP software package. These classifiers were used to predict ApicoTPs as described in this section.

Many proteins expressed in the genomes of *P. falciparum*, *P. yoelii*, *B. bovis*, and *T. gondii* are predicted to contain SPs. The cardinality of these proteins for each organism, excluding the ones that are used for training and testing, is listed in Table 5. The number of proteins predicted to be ApicoTPs by ApicoAP is also listed in Table 5.

**Table 5:** ApicoAP predictions for SP-containing *P. falciparum*, *B. bovis*, *T. gondii*, and *P. yoelii* proteins.

Organism	SP-containing protein count (excluding training data)	ApicoAP positive prediction count
*P. falciparum*	1046	542
*B. bovis*	515	194
*T. gondii*	1037	417
*P. yoelii*	1049	285

Of the 1046 SP-containing *P. falciparum* proteins, 358 are predicted to be ApicoTPs by PlasmoAP. Of these 358, 261 (261/358 = 73%) are also predicted to be ApicoTPs by ApicoAP. The remaining SP-containing *P. falciparum* proteins (1046-358 = 688) are predicted to be non-ApicoTPs by PlasmoAP. Of these 688, 407 (407/688 = 60%) are also predicted to be non-ApicoTPs by ApicoAP. This leaves 281 (688-407 = 281) that are identified as additional putative ApicoTPs by ApicoAP.

Due to a lack of prediction tools in the literature for *B. bovis*, *P. yoelii*, and *T. gondii*, we were unable to compare our prediction results against a reference. Lists of putative ApicoTPs identified by ApicoAP for the four organisms considered are available in Tables S9, S10, S11, S12.

### Optimized model parameters for ApicoAP classifiers

**Figure 2:**
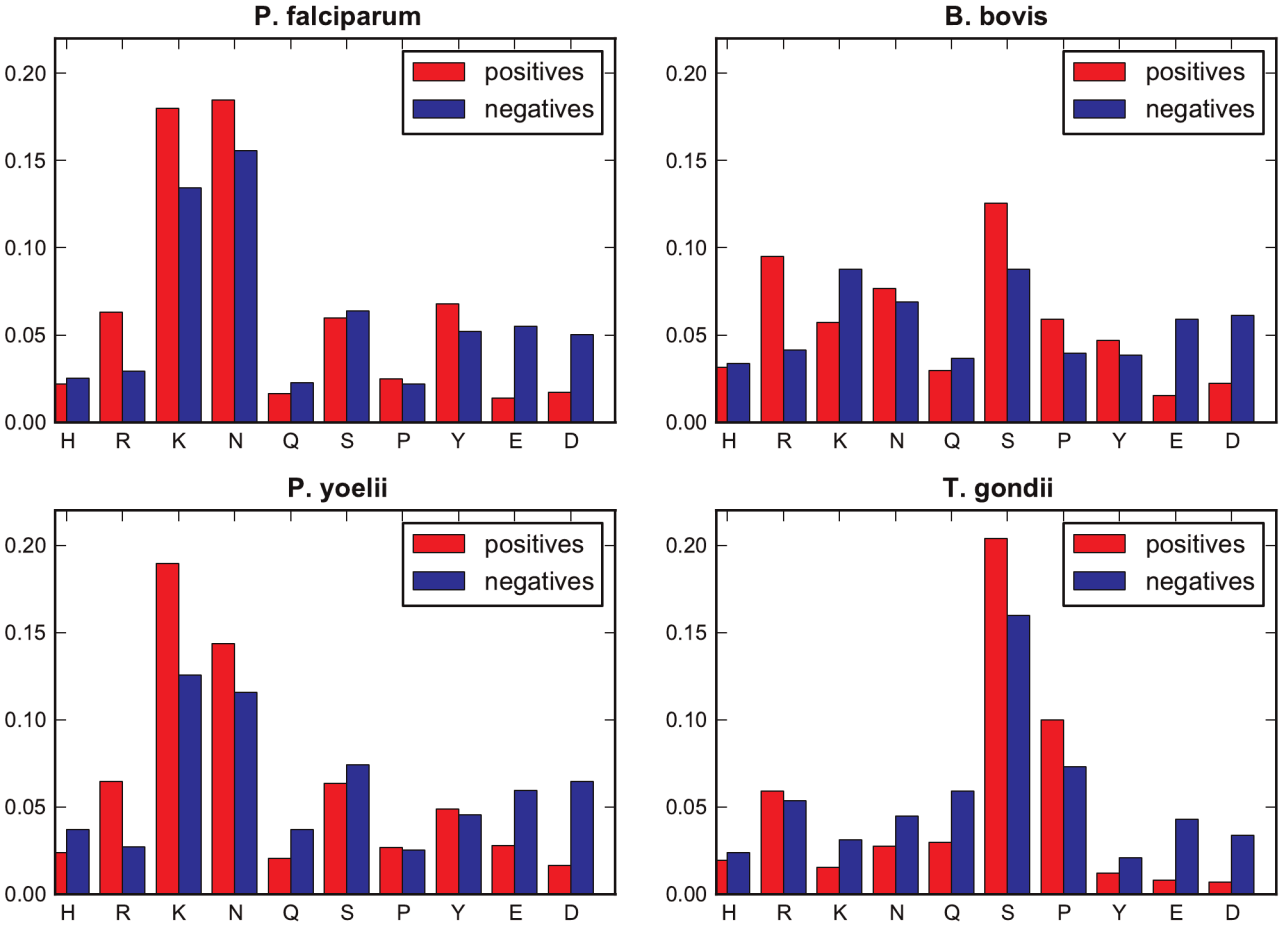
Averaged frequency distributions of preferred and avoided residues for the *p* regions of the training sequences. This figure presents the frequency distributions of *preferred* and *avoided residues* for the *p* regions of the training sequences for each organism. *p* is the contiguous sub-region with length *L_p_* in the anticipated TP region *r* that has the maximum *p-criterion* value, given by Eq. (3). Final ApicoAP classifiers are used to identify *p* regions over each sequence. Residue counts over individual *p* regions are divided by the lengths of the *p* regions, and the resulting values are averaged over positive and negative training sets for each organism.

Figure 2 presents the frequency distributions for the *preferred* and *avoided residues* within the *p* regions of the training sequences for each organism. These regions are detected by applying the final ApicoAP classifiers to the sequences. In general, weight parameter estimates are found to be proportional to the differences between the frequency of residues for positive and negative sets. For *P. falciparum*, lysine (K) seems to have the greatest effect among the amino acids contributing to the *preferred residue set score* (*PRSS*). The greatest effect on the *PRSS* for the *P. yoelii* and *B. bovis* classifiers comes from Arginine (R) and for the *T. gondii* classifier it comes from Serine (S). All these estimates seem to be consistent with the given histograms.

The estimated region length parameter *r* was found to be 60, 62, 70, and 88 for *P. falciparum*, *P. yoelii*, *B. bovis*, and *T. gondii*, respectively. The estimated length of the *p* region was found to be 31, 36, 35, and 28 for *P. falciparum*, *P. yoelii*, *B. bovis*, and *T. gondii*, respectively.

**Figure 3:**
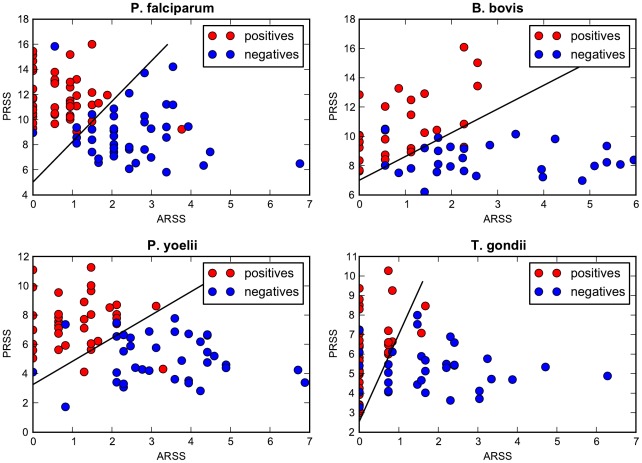
Training data mapped onto the *PRSS-ARSS* plane using final ApicoAP classifiers. This figure shows how training data are mapped onto the *PRSS-ARSS* plane when the final ApicoAP classifiers are applied. The *preferred residue set score (PRSS)* and *avoided residue set score (ARSS)* quantify the existence of *preferred residue set (PRS)* and *avoided residue set (ARS)* elements in the *p* regions of the training sequences for each organism. See Eqs. (1) and (2) for definitions. The discriminating lines are shown on each plot, where the *PRSS*-intercept of each line corresponds to the estimated limiting value *lv*, given by Eq. (4), and the slope of each line corresponds to the estimated threshold value over the *p-criterion* values, given by Eq. (3).

Figure 3 shows how training data are mapped onto the *PRSS-ARSS* plane when the final classifiers are applied. The discriminating line is shown, where the *PRSS*-intercept of this line corresponds to the estimated limiting value *lv*, given by Eq. (4), and the slope of the line corresponds to the estimated threshold value over the *p-criterion* value, given by Eq. (3). One interesting observation is that many of the *T. gondii* proteins contain *p* regions with no acidic residues, i.e. the *ARSS* is zero. Misclassifications of negative training data appear to be associated with this type of *p* region.

In addition to the content of the *p* regions presented in Figure 2 we analyzed the locations of these regions among our positive training data (with cardinality of 144). In about 55% of the sequences, the *p* region identified (with max *p-criterion* value) appears immediately after or within 5 residues of the predicted SP cleavage site. For the remaining sequences, the *p* region appears (on average) 20 residues away from the SP cleavage site. We analyzed the region between the predicted SP cleavage site and the start of the *p* region, which we refer to as the *pre-pattern* region. In order to account for SP cleavage site prediction errors, we assume a *pre-pattern* region exists when the *p* region appears 5 or more residues away from the predicted SP cleavage site. Our goal was to compare the acidic residue (D and E) frequencies of these two regions. Hypothesis testing was applied to confirm that the mean of the difference differs from zero. For this test and for all the interval estimates following, we used a p-value of 0.05. The acidic residue frequency in the *pre-pattern* region was observed to be higher than in the *p* region by 8% to 11% in 78% of these proteins. The highest and lowest differences observed were 33% and 1%, respectively.

We repeated the same analysis on a subset of our positive training data containing only the experimentally confirmed ApicoTPs (with cardinality of 70). In 43% of these, a *pre-pattern* region existed. The acidic residue frequency in the *pre-pattern* region was observed to be higher than in the *p* region by 6% to 11% in 90% of these proteins. Similar tendencies were also observed among the ApicoTPs predicted by ApicoAP.

Experimental findings for *T. gondii* transit peptides (TP) indicate that the absence of acidic residues in the N-terminal portion of the TP is important for TP fidelity, even more important than the presence of positive charge [21]. Tonkin et al. used the acyl carrier protein (ACP) from *T. gondii* in these experiments. ApicoAP identifies no *pre-pattern* region in this particular protein, which means that the *p* region is located immediately after the predicted SP cleavage site. This indicates that the prediction mechanism of ApicoAP, based entirely on the *p* region, which does not necessarily appear on the N-terminal portion of a TP, does not contradict the experimental findings.

## Discussion

The apicoplast is a unique organelle that resides in a group of eukaryotic parasites, known as Apicomplexa, which are responsible for a wide range of serious diseases among humans and livestock. As resistance to commonly used drugs increases in Apicomplexan parasites, it is important to find new drug targets. The apicoplast is an essential organelle for the survival of these parasites and, with its prokaryotic origin, is viewed as a promising drug target. The majority of apicoplast proteins are nuclear-encoded and targeted post-translationally to the apicoplast organelle. Experimental identification of apicoplast-targeted proteins (ApicoTPs) is a costly and time-consuming task. Accurate *in silico* prediction methods are needed to accelerate the identification of promising drug targets.

The computational approach available for genome-wide ApicoTP prediction, known as PlasmoAP [17], was developed to identify ApicoTPs in *P. falciparum* and, as such, application to other Apicomplexa is considered to be unreliable. We have developed an alternative computational model ApicoAP. In ApicoAP, we conduct a systematic search over a rule space using the expected prediction performance of a rule on a training set as the optimization criterion. The rule space is formalized by our parametric rule definition, and optimization is performed using a genetic algorithm. A major advantage of our approach to the genome-wide ApicoTP prediction task is that it is not restricted to a single organism but rather is customizable to different organisms for which training data are available.

Performance of ApicoAP is evaluated for labeled datasets of *P. falciparum*, *P. yoelii*, *B. bovis*, and *T. gondii* proteins, one of which is the dataset published in conjunction with PlasmoAP [17]. The evaluation utilizes cross validation, a common approach used to validate classification models. The cross-validation procedure provides an estimate of the prediction performance of a model by systematically retaining a portion of a labeled dataset and using this portion to test the model obtained using the remainder of the dataset. The expected prediction accuracies, i.e., the accuracy for unknown proteins rather than the accuracy for labeled data, for the current ApicoAp classifiers for *P. falciparum*, *P. yoelii*, *B. bovis*, and *T. gondii* are found to be 87%, 85%, 82%, and 83%, respectively. The best expected prediction accuracy is achieved using the *P. falciparum* training set, the largest of the four training sets. The larger the training data set, the more robust and accurate the resulting classifier is expected to be. With the addition of more training data, the classifiers can be updated to provide greater accuracy. While the four classifiers are specifically for use with the four species described, they may assist in the identification of potential ApicoTPs for related species when the AT-codon biases of the corresponding genomes are similar.

In this paper we present ApicoAP, the first computational model capable of identifying ApicoTPs in multiple species of Apicomplexa. In addition, we provide a user-friendly, Python-based program that includes the ApicoAP classifiers for *P. falciparum*, *P. yoelii*, *B. bovis*, and *T. gondii*. ApicoAP provides a learning framework for ApicoTP prediction based on a systematic approach to finding the rule-based classifier with the best expected prediction performance over a training set. This framework can be applied to other domains for which it is desirable to have a discriminating rule-finding process that is automated.

## Supporting Information

Table S1 **Positive training set for** ***P. falciparum.***
(DOC)

Table S2 **Positive training set for** ***P. yoelii.***
(DOC)

Table S3 **Positive training set for** ***B. bovis.***
(DOC)

Table S4 **Positive training set for** ***T. gondii.***
(DOC)

Table S5 **Negative training set for** ***P. falciparum.***
(DOC)

Table S6 **Negative training set for** ***P. yoelii.***
(DOC)

Table S7 **Negative training set for** ***B. bovis.***
(DOC)

Table S8 **Negative training set for** ***T. gondii.***
(DOC)

Table S9 **List of putative A**pico**TPs for** ***P. falciparum.***
(DOC)

Table S10 **List of putative A**pico**TPs for** ***P. yoelii.***
(DOC)

Table S11 **List of putative A**pico**TPs for** ***B. bovis.***
(DOC)

Table S12 **List of putative A**pico**TPs for** ***T. gondii.***
(DOC)
