# Supplementary material for: ApicoAP: The First Computational Model for Identifying Apicoplast-Targeted Proteins in Multiple Species of Apicomplexa
Source: PLoS One. 2012 May 4;7(5):e36598. doi: 10.1371/journal.pone.0036598 (PMC3344922; doi:10.1371/journal.pone.0036598)
Supplement: Table S1 — Positive training set for P. falciparum. (DOC) [file pone.0036598.s001.doc]

***Table S1: Positive training set for P. falciparum.***

| **Gene id** | **EuPathDB product description** | **Source** |
| --- | --- | --- |
| MAL13P1.220 | lipoate synthase, putative | Confirmed localization to Apicoplast, ApiLoc |
| PF07_0047 | AAA family ATPase, CDC48 subfamily | Confirmed localization to Apicoplast, ApiLoc |
| PF08_0063 | ClpB protein, putative | Confirmed localization to Apicoplast, ApiLoc |
| PF08_0066 | lipoamide dehydrogenase | Confirmed localization to Apicoplast, ApiLoc |
| PF08_0067 | ubiquitin | Confirmed localization to Apicoplast, ApiLoc |
| PF10_0363 | pyruvate kinase 2, putative | Confirmed localization to Apicoplast, ApiLoc |
| PF10_0407 | dihydrolipoamide acyltransferase component E2 | Confirmed localization to Apicoplast, ApiLoc |
| PF11_0256 | pyruvate dehydrogenase E1 alpha subunit | Confirmed localization to Apicoplast, ApiLoc |
| PF13_0180 | cochaperonin | Confirmed localization to Apicoplast, ApiLoc |
| PF13_0182 | ubiquitin-activating enzyme | Confirmed localization to Apicoplast, ApiLoc |
| PF14_0063 | ATP-dependent Clp protease, putative | Confirmed localization to Apicoplast, ApiLoc |
| PF14_0112 | plastid replication-repair enzyme | Confirmed localization to Apicoplast, ApiLoc |
| PF14_0381 | delta-aminolevulinic acid dehydratase | Confirmed localization to Apicoplast, ApiLoc |
| PF14_0382 | stromal-processing peptidase, putative | Confirmed localization to Apicoplast, ApiLoc |
| PF14_0498 | DER1-like protein | Confirmed localization to Apicoplast, ApiLoc |
| PF14_0641 | 1-deoxy-D-xylulose 5-phosphate reductoisomerase | Confirmed localization to Apicoplast, ApiLoc |
| PFB0385w | acyl carrier protein | Confirmed localization to Apicoplast, ApiLoc |
| PFB0505c | beta-ketoacyl-ACP synthase III | Confirmed localization to Apicoplast, ApiLoc |
| PFC0310c | ATP-dependent Clp protease proteolytic subunit | Confirmed localization to Apicoplast, ApiLoc |
| PFC0590c | DER1-like protein, putative | Confirmed localization to Apicoplast, ApiLoc |
| PFE0150c | 4-diphosphocytidyl-2c-methyl-D-erythritol kinase (CMK), putative | Confirmed localization to Apicoplast, ApiLoc |
| PFE0435c | single-strand binding protein, putative | Confirmed localization to Apicoplast, ApiLoc |
| PFE1460w | apicoplast TIC22 precursor | Confirmed localization to Apicoplast, ApiLoc |
| PFE1510c | triose phosphate transporter | Confirmed localization to Apicoplast, ApiLoc |
| PFF0360w | uroporphyrinogen III decarboxylase | Confirmed localization to Apicoplast, ApiLoc |
| PFF0730c | enoyl-acyl carrier reductase | Confirmed localization to Apicoplast, ApiLoc |
| PFF1130c | superoxide dismutase | Confirmed localization to Apicoplast, ApiLoc |
| PFI0230c | bacterial histone-like protein | Confirmed localization to Apicoplast, ApiLoc |
| PFI0380c | peptidyl deformylase | Confirmed localization to Apicoplast, ApiLoc |
| PFL0285w | targeted glyoxalase II | Confirmed localization to Apicoplast, ApiLoc |
| PFL0480w | porphobilinogen deaminase | Confirmed localization to Apicoplast, ApiLoc |
| PFL1120c | DNA gyrase subunit A | Confirmed localization to Apicoplast, ApiLoc |
| PFL1545c | chaperonin, cpn60 | Confirmed localization to Apicoplast, ApiLoc |
| PFL1915w | DNA gyrase subunit B | Confirmed localization to Apicoplast, ApiLoc |
| PFI1125c | beta-ketoacyl-acyl carrier protein reductase | Ortholog to confirmed protein TGME49_017740 (OG5_126618), [38] |
| PF10_0149 | cysteinyl-tRNA synthetase, putative | Ortholog to confirmed protein TGME49_099810 (OG5_126755), ApiLoc |
| PFF1275c | 3-oxoacyl-acyl-carrier protein synthase I/II | Ortholog to confirmed protein TGME49_093590 (OG5_126985), [38] |
| PFB0270w | iron-sulfur assembly protein, putative | Ortholog to confirmed protein TGME49_039320 (OG5_127283), [38] |
| PF14_0132 | 40S ribosomal protein S9A, putative | Ortholog to confirmed protein TGME49_018850 (OG5_127478), ApiLoc |
| PF14_0441 | pyruvate dehydrogenase E1 beta subunit | Ortholog to confirmed protein TGME49_072290 (OG5_127524), ApiLoc |
| PF08_0011 | leucine-tRNA ligase | Ortholog to confirmed protein TGME49_066730 (OG5_127675), ApiLoc |
| PF13_0066 | malonyl CoA-acyl carrier protein transacylase precursor | Ortholog to confirmed protein TGME49_025990 (OG5_127857), [38] |
| PFB0420w | 2C-methyl-D-erythritol 2,4-cyclodiphosphate synthase | Ortholog to confirmed protein TGME49_055690 (OG5_130616), [38] |
| MAL13P1.95 | ferredoxin, putative | Ortholog to confirmed protein TGME49_015070 (OG5_131654), ApiLoc |
| PFF1115w | ferredoxin NADP reductase | Ortholog to confirmed protein TGME49_098990 (OG5_133183), ApiLoc |
| PFI0810c | apicoplast Ufd1 precursor | Ortholog to confirmed protein TGME49_085700 (OG5_144501), ApiLoc |
| MAL13P1.186 | 1-deoxy-D-xylulose 5-phosphate synthase | Ortholog to confirmed protein TGME49_008820 (OG5_129068), [38] |

Note: OGx references refer to OrthoMCL-DB [32] ortholog group numbers.
