# Supplementary material for: ApicoAP: The First Computational Model for Identifying Apicoplast-Targeted Proteins in Multiple Species of Apicomplexa
Source: PLoS One. 2012 May 4;7(5):e36598. doi: 10.1371/journal.pone.0036598 (PMC3344922; doi:10.1371/journal.pone.0036598)
Supplement: Table S2 — Positive training set for P. yoelii. (DOC) [file pone.0036598.s002.doc]

***Table S2: Positive training set for P. yoelii.***

| **Gene id** | **EuPathDB product description** | **Source** |
| --- | --- | --- |
| PY00573 | dihydrolipoamide dehydrogenase | Confirmed localization to Apicoplast, ApiLoc |
| PY03846 | enoyl-acyl carrier reductase | Confirmed localization to Apicoplast, ApiLoc |
| PY02416 | 3-oxoacyl-acyl-carrier protein reductase precursor | Ortholog to confirmed protein TGME49_017740 (OG5_126618), [38] |
| PY00565 | ClpB protein | Ortholog to confirmed protein PF08_0063 (OG5_126636), ApiLoc |
| PY05364 | clpB protein | Ortholog to confirmed protein PF08_0063 (OG5_126636), ApiLoc |
| PY04452 | 3-oxoacyl-acyl-carrier protein synthase I/II | Ortholog to confirmed protein TGME49_093590 (OG5_126985), [38] |
| PY04779 | acyl carrier protein, putative | Ortholog to confirmed protein PFB0385w,TGME49_064080 (OG5_127028), ApiLoc |
| PY02505 | isocitrate dehydrogenase, NADP-dependent, putative | Ortholog to confirmed protein TGME49_066760 (OG5_127057), ApiLoc |
| PY00163 | POM1 | Ortholog to confirmed protein PF14_0112 (OG5_127218), ApiLoc |
| PY05037 | sufE protein, putative | Ortholog to confirmed protein TGME49_039320 (OG5_127283), [38] |
| PY06208 | lipoic acid synthetase, putative | Ortholog to confirmed protein MAL13P1.220 (OG5_127348), ApiLoc |
| PY05238 | SINGLE-STRAND BINDING PROTEIN | Ortholog to confirmed protein PFE0435c (OG5_127389), ApiLoc |
| PY01828 | porphobilinogen deaminase, putative | Ortholog to confirmed protein PFL0480w (OG5_127395), ApiLoc |
| PY07168 | ribosomal protein S9, putative | Ortholog to confirmed protein TGME49_018850 (OG5_127478), ApiLoc |
| PY04501 | putative ligase in lipoate biosynthesis | Ortholog to confirmed protein TGME49_115640 (OG5_127515), [38] |
| PY05492 | malonyl CoA-acyl carrier protein transacylase precursor | Ortholog to confirmed protein TGME49_025990 (OG5_127857), [38] |
| PY01738 | polypeptide deformylase, putative | Ortholog to confirmed protein PFI0380c (OG5_128237), ApiLoc |
| PY04024 | DNA topoisomerase II, putative | Ortholog to confirmed protein PFL1915w (OG5_128755), ApiLoc |
| PY00590 | putative ubiquitin-conjugating enzyme 16 | Ortholog to confirmed protein TGME49_095990 (OG5_128794), [38] |
| PY07326 | DNA gyrase subunit a-related | Ortholog to confirmed protein PFL1120c (OG5_129568), ApiLoc |
| PY05578 | 1-deoxy-D-xylulose 5-phosphate reductoisomerase | Ortholog to confirmed protein PF14_0641,TGME49_014850 (OG5_130462), ApiLoc |
| PY00321 | YgbB family, putative | Ortholog to confirmed protein TGME49_055690 (OG5_130616), [38] |
| PY04665 | 4-diphosphocytidyl-2-c-methyl-d-erythritol kinase (ec 2.7.1.148) (cmk) | Ortholog to confirmed protein PFE0150c (OG5_130885), ApiLoc |
| PY03801 | ferredoxin | Ortholog to confirmed protein TGME49_015070 (OG5_131654), ApiLoc |
| PY06273 | ferredoxin NADP reductase, putative | Ortholog to confirmed protein TGME49_098990 (OG5_133183), ApiLoc |
| PY04573 | putative dihydrolipoamide S-acetyltransferase | Ortholog to confirmed protein PF10_0407,TGME49_006610 (OG5_134960), ApiLoc |
| PY01851 | ThiF family, putative | Ortholog to confirmed protein PF13_0182 (OG5_146901), ApiLoc |
| PY00696 | hypothetical protein | Ortholog to confirmed protein TGME49_110770 (OG5_147426), [38] |
| PY00872 | hypothetical protein | Ortholog to confirmed protein TGME49_039680 (OG5_147466), [38] |
| PY01812 | phophate translocator | Ortholog to confirmed protein PFE1510c (OG5_149875), ApiLoc |
| PY04273 | hypothetical protein | Ortholog to confirmed protein TGME49_059230 (OG5_150490), [38] |
| PY02283 | hypothetical protein | Ortholog to confirmed protein PFC0590c (OG5_156784), ApiLoc |
| PY04892 | superoxide dismutase, C-terminal domain, putative | Ortholog to confirmed protein PFF1130c (OG5_158563), ApiLoc |
| PY06810 | hypothetical protein | Ortholog to confirmed protein PF14_0498 (OG5_162102), ApiLoc |

Note: OGx references refer to OrthoMCL-DB [32] ortholog group numbers.
