# Supplementary material for: ApicoAP: The First Computational Model for Identifying Apicoplast-Targeted Proteins in Multiple Species of Apicomplexa
Source: PLoS One. 2012 May 4;7(5):e36598. doi: 10.1371/journal.pone.0036598 (PMC3344922; doi:10.1371/journal.pone.0036598)
Supplement: Table S3 — Positive training set for B. bovis. (DOC) [file pone.0036598.s003.doc]

**Table S3: Positive training set for *B. bovis.***

| **Gene id** | **EuPathDB product description** | **Source** |
| --- | --- | --- |
| BBOV_III001660 | LytB protein, putative | Confirmed localization to Apicoplast, [37] |
| JN114412 | acyl carrier protein | Confirmed localization to Apicoplast, [37] |
| BBOV_III005960 | tRNA methyl transferase family protein | Confirmed localization to Apicoplast, [35] |
| BBOV_IV010640 | glutamyl-tRNA synthetase, putative | Confirmed localization to Apicoplast, personal communication with Lau AO. |
| BBOV_III010130 | pyruvate kinase family protein | Ortholog to confirmed protein PF10_0363 (OG5_144357), ApiLoc |
| BBOV_II001120 | delta-aminolevulinic acid dehydratase, putative | Ortholog to confirmed protein PF14_0381 (OG5_127486), ApiLoc |
| BBOV_IV003640 | DNA gyrase A subunit, putative | Ortholog to confirmed protein PFL1120c (OG5_129568), ApiLoc |
| BBOV_IV007010 | chaperonin cpn60, putative | Ortholog to confirmed protein PFL1545c (OG5_135175), ApiLoc |
| BBOV_IV011850 | DNA gyrase subunit B, putative | Ortholog to confirmed protein PFL1915w (OG5_128755), ApiLoc |
| BBOV_II005080 | chain A of Ferredoxin, putative | Ortholog to confirmed protein TGME49_015070 (OG5_131654), ApiLoc |
| BBOV_III002890 | conserved hypothetical protein | Ortholog to confirmed protein TGME49_027970 (OG5_130206), [38] |
| BBOV_IV002370 | leucyl-tRNA synthetase, putative | Ortholog to confirmed protein TGME49_066730 (OG5_127675), ApiLoc |
| BBOV_IV011730 | ubiquitin fusion degradation protein UFD1, putative | Ortholog to confirmed protein TGME49_085700 (OG5_144501), ApiLoc |
| BBOV_II006740 | ubiquitin-conjugating enzyme E2 | Ortholog to confirmed protein TGME49_095990 (OG5_128794), [38] |
| BBOV_II002190 | hypothetical protein | Ortholog to confirmed protein TGME49_112110 (OG5_128012), ApiLoc |
| BBOV_IV001700 | cell division cycle protein ATPase, putative | Ortholog to confirmed protein TGME49_121640 (OG5_126926), ApiLoc |
| BBOV_I004960 | triose phosphate/phosphate translocator, putative | Ortholog to confirmed proteins PFE0410w and TGME49_061070 (OG5_128059), ApiLoc |
| BBOV_IV003350 | cysteine desulfurase | Predicted as ApicoTP because of the involvement in Isoprenoid and Fe-S cluster biosynthesis pathways by [33] |
| BBOV_II006930 | gcpE protein | Predicted as ApicoTP because of the involvement in Isoprenoid and Fe-S cluster biosynthesis pathways by [33] |
| BBOV_I000970 | DnaJ domain containing protein | DnaJ homologue of Plasmodium falciparum interacts with replication ori of the apicoplast genome [34] |
| BBOV_III007840 | dnaJ domain containing protein | DnaJ homologue of Plasmodium falciparum interacts with replication ori of the apicoplast genome [34] |
| BBOV_III005720 | tRNA pseudouridine synthase, putative | Similar to PF10_0175 which is annotated as ApicoTP in geneDB |
| BBOV_I004270 | ATP-dependent Clp protease adaptor protein ClpS domain containing protein | Similar to PFC0310c which is a confirmed ApicoTP, ApiLoc |
| BBOV_IV000910 | S-adenosyl methyltransferase, putative | Similar to PFL1775c which is annotated as ApicoTP in geneDB |
| BBOV_IV001460 | adrenodoxin-type ferredoxin, putative | Structurally similar to confirmed TGME49_015070, ApiLoc |
| BBOV_I004040 | conserved hypothetical protein | Structurally similar to PFB0390w which is annotated as ApicoTP in geneDB |
| BBOV_I003640 | DnaK family domain containing protein | TP often has DnaK binding sites [17] |
| BBOV_IV004710 | translation elongation factor G (EF-G), putative | Similar protein is suggested to be a likely ApicoTP by [36] |

Note: OGx references refer to OrthoMCL-DB [32] ortholog group numbers.
