# Supplementary material for: ApicoAP: The First Computational Model for Identifying Apicoplast-Targeted Proteins in Multiple Species of Apicomplexa
Source: PLoS One. 2012 May 4;7(5):e36598. doi: 10.1371/journal.pone.0036598 (PMC3344922; doi:10.1371/journal.pone.0036598)
Supplement: Table S4 — Positive training set for T. gondii. (DOC) [file pone.0036598.s004.doc]

***Table S4: Positive training set for T. gondii.***

| **Gene id** | **EuPathDB product description** | **Source** |
| --- | --- | --- |
| TGME49_009710 | 50S ribosomal protein L28, putative | Confirmed localization to Apicoplast, ApiLoc |
| TGME49_121570 | (3R)-hydroxymyristoyl ACP dehydrase, putative | Confirmed localization to Apicoplast, ApiLoc |
| TGME49_021320 | acetyl-CoA carboxylase, putative | Confirmed localization to Apicoplast, ApiLoc |
| TGME49_022020 | phosphoglycerate kinase, putative | Confirmed localization to Apicoplast, ApiLoc |
| TGME49_098990 | ferredoxin NADP+ oxidoreductase, putative | Confirmed localization to Apicoplast, ApiLoc |
| TGME49_066760 | isocitrate dehydrogenase, putative | Confirmed localization to Apicoplast, ApiLoc |
| TGME49_105980 | dihydrolipoyl dehydrogenase protein, putative | Confirmed localization to Apicoplast, ApiLoc |
| TGME49_106060 | hypothetical protein | Confirmed localization to Apicoplast, ApiLoc |
| TGME49_006610 | biotin requiring domain-containing protein / 2-oxo acid dehydrogenases acyltransferase catalytic domain-containing protein | Confirmed localization to Apicoplast, ApiLoc |
| TGME49_064080 | acyl carrier protein | Confirmed localization to Apicoplast, ApiLoc |
| TGME49_059260 | cell division protein, putative | Confirmed localization to Apicoplast, ApiLoc |
| TGME49_072290 | pyruvate dehydrogenase E1 beta subunit, putative | Confirmed localization to Apicoplast, ApiLoc |
| TGME49_026730 | aconitate hydratase, putative | Confirmed localization to Apicoplast, ApiLoc |
| TGME49_116330 | iron-containing superoxide dismutase | Confirmed localization to Apicoplast, ApiLoc |
| TGME49_018850 | ribosomal protein S9, putative | Confirmed localization to Apicoplast, ApiLoc |
| TGME49_051930 | enoyl-acyl carrier reductase | Confirmed localization to Apicoplast, ApiLoc |
| TGME49_093590 | 3-oxoacyl-[acyl-carrier-protein] synthase, putative | Confirmed localization to Apicoplast, [38] |
| TGME49_053900 | delta-aminolevulinic acid dehydratase, putative | Confirmed localization to Apicoplast, [38] |
| TGME49_091670 | RNA helicase, putative | Confirmed localization to Apicoplast, [38] |
| TGME49_086050 | hypothetical protein | Confirmed localization to Apicoplast, [38] |
| TGME49_040600 | TCP-1/cpn60 chaperonin family protein, putative | Confirmed localization to Apicoplast, [38] |
| TGME49_002440 | hypothetical protein | Confirmed localization to Apicoplast, [38] |
| TGME49_059230 | hypothetical protein | Confirmed localization to Apicoplast, [38] |
| TGME49_072390 | hypothetical protein | Confirmed localization to Apicoplast, [38] |
| TGME49_071420 | porphobilinogen deaminase, putative | Confirmed localization to Apicoplast, [38] |
| TGME49_027970 | DNA-binding protein HU, putative | Confirmed localization to Apicoplast, [38] |
| TGME49_025990 | malonyl CoA-acyl carrier protein transacylase, putative | Confirmed localization to Apicoplast, [38] |
| TGME49_110770 | hypothetical protein, conserved | Confirmed localization to Apicoplast, [38] |
| TGME49_114890 | ubiquitin-activating enzyme E1, putative | Confirmed localization to Apicoplast, [38] |
| TGME49_017740 | oxoacyl-ACP reductase, putative | Confirmed localization to Apicoplast, [38] |
| TGME49_002580 | heat shock protein, putative | Ortholog to confirmed protein PF08_0063 (OG5_126636), ApiLoc |
| TGME49_082200 | clpB protein, putative | Ortholog to confirmed protein PF08_0063 (OG5_126636), ApiLoc |
| TGME49_016700 | peptide deformylase, putative | Ortholog to confirmed protein PFI0380c (OG5_128237), ApiLoc |
| TGME49_003270 | ATP-dependent Clp protease proteolytic subunit, putative | Similar to confirmed PFC0310c, ApiLoc |
| TGME49_106550 | 4-diphosphocytidyl-2-C-methyl-D-erythritol kinase, putative | Similar to confirmed PFE0150c, ApiLoc |

Note: OGx references refer to OrthoMCL-DB [32] ortholog group numbers.
