# Supplementary material for: ApicoAP: The First Computational Model for Identifying Apicoplast-Targeted Proteins in Multiple Species of Apicomplexa
Source: PLoS One. 2012 May 4;7(5):e36598. doi: 10.1371/journal.pone.0036598 (PMC3344922; doi:10.1371/journal.pone.0036598)
Supplement: Table S5 — Negative training set for P. falciparum. (DOC) [file pone.0036598.s005.doc]

***Table S5: Negative training set for P. falciparum.***

| **Gene id** | **EuPathDB product description** | **Source** |
| --- | --- | --- |
| PF10_0303 | 25 kDa ookinete surface antigen precursor | Confirmed localization: parasite plasma membrane, vesicle, ApiLoc |
| PFB0400w | 6-cysteine protein | Confirmed localization: cytoplasm,parasite plasma membrane, ApiLoc |
| PF13_0248 | 6-cysteine protein | Confirmed localization: parasite plasma membrane, ApiLoc |
| PFE0395c | 6-cysteine protein | Confirmed localization: merozoite surface , ApiLoc |
| PFF0615c | 6-cysteine protein | Confirmed localization: parasite plasma membrane, ApiLoc |
| PFB0405w | 6-cysteine protein | Confirmed localization: parasite plasma membrane, ApiLoc |
| PF13_0247 | 6-cysteine protein | Confirmed localization: parasite plasma membrane, ApiLoc |
| PFD0240c | 6-cysteine protein | Confirmed localization: parasite plasma membrane, ApiLoc |
| PFC0210c | circumsporozoite (CS) protein | Confirmed localization: sporozoite surface, parasite plasma membrane, ApiLoc |
| PF11_0224 | circumsporozoite-related antigen | Confirmed localization: parasitophorous vacuole lumen, erythrocyte cytoplasmic structure, maurer's cleft, ApiLoc |
| MAL7P1.92 | cysteine repeat modular protein 2 | Confirmed localization: sporozoite surface, maurer's cleft, parasite plasma membrane, ApiLoc |
| PF13_0338 | cysteine-rich surface protein | Confirmed localization: merozoite surface, parasite plasma membrane, ApiLoc |
| PF10_0348 | duffy binding-like merozoite surface protein | Confirmed localization: merozoite surface, parasite plasma membrane, ApiLoc |
| MAL13P1.60 | erythrocyte binding antigen-140 | Confirmed localization: microneme, ApiLoc |
| MAL7P1.176 | erythrocyte binding antigen-175 | Confirmed localization: rhoptry neck, microneme, ApiLoc |
| PFA0125c | erythrocyte binding antigen-181 | Confirmed localization: microneme, ApiLoc |
| PFB0095c | erythrocyte membrane protein 3 | Confirmed localization: exported, maurer's cleft,erythrocyte cytoplasm, parasitophorous vacuole, ApiLoc |
| PFI1445w | high molecular weight rhoptry protein 2 | Confirmed localization: rhoptry, parasite plasma membrane , ApiLoc |
| PFI0265c | high molecular weight rhoptry protein 3 | Confirmed localization: erythrocyte plasma membrane, rhoptry, ApiLoc |
| PF14_0067 | LCCL domain-containing protein | Confirmed localization: exported,parasite plasma membrane, ApiLoc |
| PFA0445w | LCCL domain-containing protein | Confirmed localization: parasite plasma membrane, cell poles, ApiLoc |
| PF14_0532 | LCCL domain-containing protein | Confirmed localization: parasite plasma membrane, ApiLoc |
| PF14_0723 | LCCL domain-containing protein | Confirmed localization: parasite plasma membrane, ApiLoc |
| PFI0185w | LCCL domain-containing protein | Confirmed localization: parasite plasma membrane, ApiLoc |
| PF14_0491 | LCCL-like protein | Confirmed localization: parasite plasma membrane, cell poles, ApiLoc |
| PF11_0486 | merozoite adhesive erythrocytic binding protein | Confirmed localization: parasite plasma membrane, rhoptry, ApiLoc |
| PF10_0352 | merozoite surface protein | Confirmed localization: merozoite surface, microneme, ApiLoc |
| PF10_0355 | merozoite surface protein | Confirmed localization: merozoite surface , ApiLoc |
| PFI1475w | merozoite surface protein 1 | Confirmed localization: merozoite surface, parasite plasma membrane, ApiLoc |
| PFF0995c | merozoite surface protein 10 | Confirmed localization: parasite plasma membrane , rhoptry, ApiLoc |
| PF10_0345 | merozoite surface protein 3 | Confirmed localization: merozoite surface, parasite plasma membrane, ApiLoc |
| PFB0305c-a | merozoite surface protein 5 | Confirmed localization: parasite plasma membrane, ApiLoc |
| PF13_0197 | merozoite surface protein 7 precursor | Confirmed localization: cytoplasm,parasite plasma membrane, ApiLoc |
| PFE0120c | merozoite surface protein 8,ring-stage membrane protein 1 | Confirmed localization: food vacuole, parasite plasma membrane, ApiLoc |
| PFL1385c | merozoite surface protein 9 | Confirmed localization: parasite plasma membrane, parasitophorous vacuole, ApiLoc |
| PF10_0281 | merozoite TRAP-like protein | Confirmed localization: microneme, merozoite surface, ApiLoc |
| PFL2405c | osmiophilic body protein | Confirmed localization: osmiophilic body , ApiLoc |
| PFC0435w | parasite-infected erythrocyte surface protein | Confirmed localization: cytoplasm,exported, ApiLoc |
| PFE0060w | parasite-infected erythrocyte surface protein | Confirmed localization: maurer's cleft, ApiLoc |
| PF11_0302 | parasitophorous vacuolar protein 1 | Confirmed localization: maurer's cleft, parasitophorous vacuole, ApiLoc |
| PFA0680c | Pfmc-2TM Maurer's cleft two transmembrane protein | Confirmed localization: maurer's cleft, parasitophorous vacuole, ApiLoc |
