# Supplementary material for: ApicoAP: The First Computational Model for Identifying Apicoplast-Targeted Proteins in Multiple Species of Apicomplexa
Source: PLoS One. 2012 May 4;7(5):e36598. doi: 10.1371/journal.pone.0036598 (PMC3344922; doi:10.1371/journal.pone.0036598)
Supplement: Table S6 — Negative training set for P. yoelii. (DOC) [file pone.0036598.s006.doc]

***Table S6: Negative training set for P. yoelii.***

| **Gene id** | **EuPathDB product description** | **Source** |
| --- | --- | --- |
| PY00204 | hypothetical protein | Confirmed localization: apical, ApiLoc |
| PY04858 | ookinete protein-related | Confirmed localization: apical, ApiLoc |
| PY01365 | rhoptry protein | Confirmed localization: apical, ApiLoc |
| PY03052 | sporozoite surface protein 2 precursor | Confirmed localization: apical, microneme, salivary gland sporozoite, ApiLoc |
| PY00522 | 28 kDa ookinete surface protein | Confirmed localization: cytoplasm,parasite plasma membrane, ApiLoc |
| PY05977 | erythrocyte binding protein | Confirmed localization: merozoite surface, rhoptry, ApiLoc |
| PY01581 | apical membrane antigen-1 | Confirmed localization: microneme, rhoptry, ApiLoc |
| PY05748 | merozoite surface protein 1 precursor | Confirmed localization: parasite plasma membrane, ApiLoc |
| PY04421 | U43539 hepatocyte erythrocyte protein 17 kDa | Confirmed localization: parasitophorous vacuole, ApiLoc |
| PY04499 | hypothetical protein | Confirmed localization: parasitophorous vacuole, ApiLoc |
| PY02159 | Drosophila melanogaster CG15040 gene product | Confirmed localization: rhoptry neck, apical, ApiLoc |
| PY05001 | heat shock protein | Ortholog to confirmed nonApicoTP PFI0875w (OG5_126588), ApiLoc |
| PY07382 | hypothetical protein | Ortholog to MAL7P1.119 (OG5_160791) that is found to localize to apical, ApiLoc |
| PY00143 | hypothetical protein | Ortholog to MAL7P1.208 (OG5_153639) that is found to localize to rhoptry, endoplasmic reticulum and golgi apparatus , ApiLoc |
| PY00337 | cysteine repeat modular protein 1 PbCRM1 | Ortholog to MAL7P1.92 (OG5_131558) that is found to localize to sporozoite surface, maurer's cleft, parasite plasma membrane, ApiLoc |
| PY05272 | cysteine repeat modular protein 2 PbCRM2-related | Ortholog to MAL7P1.92 (OG5_131558) that is found to localize to sporozoite surface, maurer's cleft, parasite plasma membrane, ApiLoc |
| PY01222 | subtilisin-like protease 2 | Ortholog to PF11_0381 (OG5_149511) that is found to localize to apical, dense granule, ApiLoc |
| PY01071 | multidomain scavenger receptor protein PbSR precursor | Ortholog to PF14_0067 (OG5_135032) that is found to localize to parasite plasma membrane, ApiLoc |
| PY06813 | hypothetical protein | Ortholog to PF14_0495,TGME49_100100 (OG5_142870) that are found to localize to rhoptry neck, apical, ApiLoc |
| PY05090 | LCCL domain-containing protein CCP2-related | Ortholog to PF14_0532 (OG5_141742) that is found to localize to parasite plasma membrane, ApiLoc |
| PY05554 | LCCL domain-containing protein CCP2 | Ortholog to PF14_0723 (OG5_139606) that is found to localize to parasite plasma membrane, ApiLoc |
| PY01624 | hypothetical protein | Ortholog to PFA0445w,PF14_0491 (OG5_135662) that are found to localize to parasite plasma membrane, cell poles, ApiLoc |
| PY06758 | hypothetical protein | Ortholog to PFA0445w,PF14_0491 (OG5_135662) that are found to localize to parasite plasma membrane, cell poles, ApiLoc |
| PY00293 | Papain family cysteine protease, putative | Ortholog to PFB0335c,PFB0340c,PFB0345c (OG5_132467) that are found to localize to parasitophorous vacuole, ApiLoc |
| PY03918 | RAP2, putative | Ortholog to PFE0080c (OG5_162090) that is found to localize to rhoptry, ApiLoc |
| PY04329 | PfSUB-1 | Ortholog to PFE0370c (OG5_138369) that is found to localize to dense granule, exoneme, ApiLoc |
| PY01154 | hypothetical protein | Ortholog to PFI0185w (OG5_162072) that is found to localize to parasite plasma membrane, ApiLoc |
| PY05296 | hypothetical protein | Ortholog to PFI1445w (OG5_162079) that is found to localize to rhoptry, parasite plasma membrane, ApiLoc |
| PY00008 | chitinase | Ortholog to PFL2510w (OG5_141486) that is found to localize to zygote remnant , ApiLoc |
| PY00357 | hypothetical protein | Ortholog to TGME49_067680 (OG5_126619) that is found to localize to microneme, ApiLoc |
| PY02148 | merozoite surface protein 7 precursor | merozoite surface protein |
| PY02883 | merozoite surface protein-9 precursor, putative | merozoite surface protein |
| PY05054 | rhoptry protein | rhoptry associated protein |
| PY00649 | rhoptry protein | rhoptry associated protein |
| PY04438 | rhoptry protein-related | rhoptry associated protein |
| PY00622 | rhoptry associated protein 1 | rhoptry associated protein |

Note: OGx references refer to OrthoMCL-DB [32] ortholog group numbers.
