# Supplementary material for: ApicoAP: The First Computational Model for Identifying Apicoplast-Targeted Proteins in Multiple Species of Apicomplexa
Source: PLoS One. 2012 May 4;7(5):e36598. doi: 10.1371/journal.pone.0036598 (PMC3344922; doi:10.1371/journal.pone.0036598)
Supplement: Table S7 — Negative training set for B. bovis. (DOC) [file pone.0036598.s007.doc]

***Table S7: Negative training set for B. bovis.***

| **Gene id** | **EuPathDB product description** | **Source** |
| --- | --- | --- |
| BBOV_II002650 | thrombospondin-related anonymous protein | Confirmed localization: apical, ApiLoc |
| BBOV_IV009860 | rhoptry-associated protein 1 (RAP-1) | Confirmed localization: erythrocyte cytoplasm, cytoplasm, nucleus and parasitophorous vacuole membrane, ApiLoc |
| BBOV_IV005390 | spherical body protein 4 SBP4 | Confirmed localization: erythrocyte cytoplasm, spherical body organelle, ApiLoc |
| BBOV_II002880 | 85 kDa protein | Confirmed localization: erythrocyte membrane, spherical body organelle, ApiLoc |
| BBOV_II000740 | Spherical Body Protein 2 (SBP2) | Confirmed localization: host erythrocyte membrane, spherical body organelle, ApiLoc |
| BBOV_I004210 | spherical body protein 3 | Confirmed localization: host erythrocyte membrane, spherical body organelle, ApiLoc |
| BBOV_I002990 | merozoite surface antigen-2b (MSA-2b) | Confirmed localization: parasite plasma membrane, ApiLoc |
| BBOV_I003000 | merozoite surface antigen-2a2 (MSA-2a2) | Confirmed localization: parasite plasma membrane, ApiLoc |
| BBOV_I003010 | merozoite surface antigen-2a1 (MSA-2a1) | Confirmed localization: parasite plasma membrane, ApiLoc |
| BBOV_I003020 | merozoite surface antigen-2c (MSA-2c) | Confirmed localization: parasite plasma membrane, ApiLoc |
| BBOV_I003060 | merozoite surface antigen-1 (MSA-1) | Confirmed localization: parasite plasma membrane, ApiLoc |
| BBOV_III007800 | heat shock protein 70 precursor, putative | Ortholog to confirmed nonApicoTP PFI0875w (OG5_126588), ApiLoc |
| BBOV_III008750 | tRNA synthetases class I (W and Y) family protein | Ortholog to confirmed nonApicoTP TGME49_054110 (OG5_127531), ApiLoc |
| BBOV_I002740 | 200 kDa antigen p200 | Ortholog to PF11_0486 (OG5_126854) that is found to localize to apical, parasite plasma membrane, rhoptry, ApiLoc |
| BBOV_I001630 | membrane protein, putative | Ortholog to PF14_0495,TGME49_100100 (OG5_142870) that are found to localize to rhoptry neck, ApiLoc |
| BBOV_II003700 | LCCL domain-containing protein CCP2, putative | Ortholog to PF14_0532 (OG5_141742) that is found to localize to parasite plasma membrane, ApiLoc |
| BBOV_III006360 | LCCL domain containing protein | Ortholog to PF14_0723 (OG5_139606) that is found to localize to parasite plasma membrane, ApiLoc |
| BBOV_III007200 | conserved hypothetical protein | Ortholog to PFA0445w,PF14_0491 (OG5_135662) that are found to localize to parasite plasma membrane, ApiLoc |
| BBOV_II006080 | subtilisin-like protein, putative | Ortholog to PFE0370c (OG5_138369) that is found to localize to dense granule, exoneme, ApiLoc |
| BBOV_II006100 | rhomboid 4 | Ortholog to TGME49_068590 (OG5_135731) that is found to localize to parasite plasma membrane, ApiLoc |
| BBOV_II000170 | cathepsin C precursor, putative | Ortholog to TGME49_089620 (OG5_130494) that is found to localize to dense granule, ApiLoc |
| BBOV_IV009870 | rhoptry-associated protein 1 (RAP-1) | rhoptry-associated protein |
| BBOV_I005190 | variant erythrocyte surface antigen-1, alpha subunit | variant erythrocyte surface antigen |
| BBOV_II000030 | variant erythrocyte surface antigen-1, alpha subunit | variant erythrocyte surface antigen |
| BBOV_III000040 | variant erythrocyte surface antigen-1, alpha subunit | variant erythrocyte surface antigen |
| BBOV_III001280 | variant erythrocyte surface antigen-1, alpha subunit | variant erythrocyte surface antigen |
| BBOV_IV007910 | variant erythrocyte surface antigen-1, alpha subunit | variant erythrocyte surface antigen |
| BBOV_IV007940 | variant erythrocyte surface antigen-1, alpha subunit | variant erythrocyte surface antigen |
| BBOV_I005650 | variant erythrocyte surface antigen-1, putative | variant erythrocyte surface antigen |

Note: OGx references refer to OrthoMCL-DB [32] ortholog group numbers.
