# Supplementary material for: ApicoAP: The First Computational Model for Identifying Apicoplast-Targeted Proteins in Multiple Species of Apicomplexa
Source: PLoS One. 2012 May 4;7(5):e36598. doi: 10.1371/journal.pone.0036598 (PMC3344922; doi:10.1371/journal.pone.0036598)
Supplement: Table S8 — Negative training set for T. gondii. (DOC) [file pone.0036598.s008.doc]

***Table S8: Negative training set for*** T. gondii.

| **Gene id** | **EuPathDB product description** | **Source** |
| --- | --- | --- |
| TGME49_027620 | 28 kDa antigen | Confirmed localization: dense granule, ApiLoc |
| TGME49_089620 | cathepsin C | Confirmed localization: dense granule, ApiLoc |
| TGME49_093770 | chitinase class I, putative | Confirmed localization: microneme, ApiLoc |
| TGME49_070240 | cyst matrix protein | Confirmed localization: parasitophorous vacuole, cyst matrix, oocyst wall, ApiLoc |
| TGME49_049670 | cysteine proteinase, putative | Confirmed localization: endosomal vacuole, parasitophorous vacuole, rhoptry, ApiLoc |
| TGME49_070250 | dense granule protein 1 / major antigenp24 | Confirmed localization: oocyst wall, sporozoite parasitophorous vacuole, dense granule, ApiLoc |
| TGME49_027280 | dense granule protein 3 | Confirmed localization: dense granule, oocyst wall, parasitophorous vacuole, ApiLoc |
| TGME49_086450 | dense granule protein 5 precursor | Confirmed localization: parasitophorous vacuole membrane, oocyst wall, dense granule, ApiLoc |
| TGME49_003310 | dense granule protein 7 | Confirmed localization: dense granule, ApiLoc |
| TGME49_054720 | dense granule protein GRA8 | Confirmed localization: dense granule, parasitophorous vacuole, ApiLoc |
| TGME49_051540 | GRA9 protein, putative | Confirmed localization: dense granule, parasitophorous vacuole, ApiLoc |
| TGME49_111720 | heat shock protein 70, putative | Confirmed localization: nuclear envelope, endoplasmic reticulum, ApiLoc |
| TGME49_050710 | microneme protein 10 | Confirmed localization: microneme, ApiLoc |
| TGME49_045490 | microneme protein 8 | Confirmed localization: microneme,rhoptry, ApiLoc |
| TGME49_091890 | microneme protein MIC1 | Confirmed localization: microneme, ApiLoc |
| TGME49_004530 | microneme protein MIC11 | Confirmed localization: microneme, ApiLoc |
| TGME49_119560 | microneme protein MIC3 | Confirmed localization: microneme, parasite plasma membrane, ApiLoc |
| TGME49_008030 | microneme protein MIC4 | Confirmed localization: microneme, ApiLoc |
| TGME49_018520 | microneme protein MIC6 | Confirmed localization: microneme, ApiLoc |
| TGME49_060190 | microneme protein, putative | Confirmed localization: sub-apical, ApiLoc |
| TGME49_067680 | microneme protein, putative | Confirmed localization: microneme, ApiLoc |
| TGME49_077080 | microneme TgMIC5 protein | Confirmed localization: microneme, ApiLoc |
| TGME49_009610 | oocyst wall protein COWP, putative | Confirmed localization: oocyst wall , ApiLoc |
| TGME49_004420 | oocyst wall protein COWP, putative | Confirmed localization: oocyst wall , ApiLoc |
| TGME49_068310 | oocyst wall protein, putative | Confirmed localization: oocyst wall, ApiLoc |
| TGME49_068590 | rhomboid-like protease 4 | Confirmed localization: parasite plasma membrane, ApiLoc |
| TGME49_027810 | Rhoptry kinase family protein ROP11 (incomplete catalytic triad) | Confirmed localization: rhoptry, ApiLoc |
| TGME49_062730 | Rhoptry kinase family protein ROP16 | Confirmed localization: rhoptry, ApiLoc |
| TGME49_005250 | Rhoptry kinase family protein ROP18 | Confirmed localization: rhoptry, cytoplasmic vesicle, parasitophorous vacuole, ApiLoc |
| TGME49_108080 | Rhoptry kinase family protein ROP5 (incomplete catalytic triad) | Confirmed localization: rhoptry, ApiLoc |
| TGME49_015770 | Rhoptry kinase family protein ROP8 (incomplete catalytic triad) | Confirmed localization: rhoptry, ApiLoc |
| TGME49_100100 | rhoptry neck protein 2 | Confirmed localization: host cell plasma membrane, rhoptry neck, ApiLoc |
| TGME49_109590 | rhoptry protein, putative | Confirmed localization: rhoptry, parasitophorous vacuole, ApiLoc |
