# Supplementary material for: ApicoAP: The First Computational Model for Identifying Apicoplast-Targeted Proteins in Multiple Species of Apicomplexa
Source: PLoS One. 2012 May 4;7(5):e36598. doi: 10.1371/journal.pone.0036598 (PMC3344922; doi:10.1371/journal.pone.0036598)
Supplement: Table S9 — List of putative ApicoTPs for P. falciparum. (DOC) [file pone.0036598.s009.doc]

***Table S9: List of putative ApicoTPs for P. falciparum.***

| **Gene id** | **EuPathDB product description** | **Gene id** | **EuPathDB product description** |
| --- | --- | --- | --- |
| MAL13P1.106 | probable protein, unknown function | PFA0555c | UMP-CMP kinase, putative |
| MAL13P1.111 | ATP-dependent Clp protease adaptor protein ClpS, putative | PFA0580c | TatD-like deoxyribonuclease, putative |
| MAL13P1.156 | conserved Plasmodium protein, unknown function | PFA0670c | Plasmodium exported protein (hyp8), unknown function |
| MAL13P1.160 | conserved Plasmodium protein, unknown function | PFA0685c | Plasmodium exported protein (hyp4), unknown function |
| MAL13P1.161 | conserved Plasmodium protein, unknown function | PFA0690w | Plasmodium exported protein, unknown function, pseudogene |
| MAL13P1.171 | transmembrane protein Tmp21 homologue, putative | PFA0700c | Plasmodium exported protein (hyp10), unknown function |
| MAL13P1.196 | serine/threonine protein kinase, putative | PFA0705c | stevor, pseudogene |
| MAL13P1.217 | conserved Plasmodium protein, unknown function | PFA0715c | Plasmodium exported protein (hyp7), unknown function |
| MAL13P1.22 | DNA ligase I | PFA0735w | Plasmodium exported protein (PHISTa), unknown function |
| MAL13P1.221 | aspartate carbamoyltransferase | PFA0750w | stevor |
| MAL13P1.225 | thioredoxin 2 | PFB0025c | stevor |
| MAL13P1.227 | ubiquitin conjugating enzyme | PFB0050c | stevor, pseudogene |
| MAL13P1.25 | conserved Plasmodium protein, unknown function | PFB0055c | rifin |
| MAL13P1.251 | conserved Plasmodium protein, unknown function | PFB0065w | stevor |
| MAL13P1.254 | conserved Plasmodium protein, unknown function | PFB0090c | RESA-like protein with PHIST and DnaJ domains |
| MAL13P1.255 | N6-adenine-specific methylase, putative | PFB0100c | knob-associated histidine-rich protein |
| MAL13P1.262 | conserved Plasmodium protein, unknown function | PFB0120w | early transcribed membrane protein 2 |
| MAL13P1.281 | glutamate--tRNA ligase, putative | PFB0140w | zinc finger protein, putative |
| MAL13P1.285 | patatin-like phospholipase, putative | PFB0180w | 5'-3' exonuclease, N-terminal resolvase-like domain, putative |
| MAL13P1.299 | conserved protein, unknown function | PFB0475c | conserved Plasmodium protein, unknown function |
| MAL13P1.319 | indole-3-glycerol-phosphate synthase, putative | PFB0485c | conserved Plasmodium protein, unknown function |
| MAL13P1.320 | conserved Plasmodium membrane protein, unknown function | PFB0680w | rhoptry neck protein 6 |
| MAL13P1.324 | aldo-keto reductase, putative | PFB0685c | acyl-CoA synthetase, PfACS9 |
| MAL13P1.335 | phosphatidylserine synthase I, putative | PFB0695c | acyl-CoA synthetase, PfACS8 |
| MAL13P1.42 | recombinase, putative | PFB0725c | zinc finger protein, putative |
| MAL13P1.461 | probable protein, unknown function | PFB0760w | MtN3-like protein |
| MAL13P1.485 | acyl-CoA synthetase, PfACS4 | PFB0770c | conserved Plasmodium membrane protein, unknown function |
| MAL13P1.505 | stevor | PFB0790c | conserved Plasmodium membrane protein, unknown function |
| MAL13P1.540 | heat shock protein 70, putative | PFB0855c | apicoplast RNA methyltransferase precursor, putative |
| MAL13P1.56 | M1-family alanyl aminopeptidase | PFB0890c | pseudouridine synthase, putative |
| MAL13P1.61 | Plasmodium exported protein (hyp8), unknown function | PFB0905c | Plasmodium exported protein (PHISTc), unknown function |
| MAL13P1.62 | Plasmodium exported protein, unknown function | PFB0921c | Plasmodium exported protein, unknown function |
| MAL13P1.67 | methionyl-tRNA formyltransferase, putative | PFB0923c | Plasmodium exported protein, unknown function |
| MAL13P1.7 | stevor | PFB0955w | stevor |
| MAL13P1.78 | conserved Plasmodium protein, unknown function | PFB0960c | Pfmc-2TM family pseudogene |
| MAL7P1.119 | rhoptry-associated leucine zipper-like protein 1 | PFB0985c | Pfmc-2TM Maurer's cleft two transmembrane protein |
| MAL7P1.142 | conserved Plasmodium membrane protein, unknown function | PFB0990c | Plasmodium exported protein (hyp4), unknown function |
| MAL7P1.149 | conserved Plasmodium protein, unknown function | PFB1015w | rifin |
| MAL7P1.157 | conserved Plasmodium protein, unknown function | PFB1020w | stevor |
| MAL7P1.159 | 1-cys peroxiredoxin | PFB1040w | rifin |
| MAL7P1.170 | Plasmodium exported protein, unknown function | PFB1045w | erythrocyte membrane protein 1 (PfEMP1), truncated |
| MAL7P1.178 | alpha/beta hydrolase, putative | PFC0025c | stevor |
| MAL7P1.186 | var-like erythrocyte membrane protein 1 | PFC0040w | rifin |
| MAL7P1.203 | conserved Plasmodium protein, unknown function | PFC0045w | rifin |
| MAL7P1.218 | stevor | PFC0050c | acyl-CoA synthetase |
| MAL7P1.223 | stevor | PFC0055w | Plasmodium exported protein (hyp13), unknown function |
| MAL7P1.23 | RAP protein, putative | PFC0125w | ABC transporter, (TAP family), putative |
| MAL7P1.3 | Plasmodium exported protein (hyp5), unknown function | PFC0215c | conserved Plasmodium protein, unknown function |
| MAL7P1.31 | conserved Plasmodium membrane protein, unknown function | PFC0225c | elongation factor (EF-TS), putative |
| MAL7P1.310 | stevor, pseudogene | PFC0281w | conserved Plasmodium protein, unknown function |
| MAL7P1.33 | conserved Plasmodium protein, unknown function | PFC0330w | conserved Plasmodium protein, unknown function |
| MAL7P1.4 | Plasmodium exported protein (hyp4), unknown function | PFC0416w | conserved Plasmodium protein, unknown function |
| MAL7P1.43 | rifin, pseudogene | PFC0450w | conserved Plasmodium protein, unknown function |
| MAL7P1.5 | Pfmc-2TM Maurer's cleft two transmembrane protein | PFC0470w | valine-tRNA ligase, putative |
| MAL7P1.52 | rifin, pseudogene | PFC0490w | conserved Plasmodium protein, unknown function |
| MAL7P1.74 | secreted ookinete protein, putative | PFC0556c | apicoplast conserved ycf19 protein precursor, unknown function |
| MAL8P1.101 | RNA binding protein, putative | PFC0571c-a | conserved Plasmodium protein, unknown function |
| MAL8P1.110 | apicoplast ribosomal protein L33 precursor, putative | PFC0571c-b | conserved Plasmodium protein, unknown function |
| MAL8P1.140 | methionine aminopeptidase 1c, putative | PFC0575w | conserved Plasmodium protein, unknown function |
| MAL8P1.161 | Plasmodium exported protein (hyp7), unknown function | PFC0580c | conserved Plasmodium protein, unknown function |
| MAL8P1.163 | Plasmodium exported protein (PHISTa), unknown function | PFC0640w | CSP and TRAP-related protein |
| MAL8P1.18 | conserved Plasmodium protein, unknown function | PFC0670c | conserved Plasmodium protein, unknown function |
| MAL8P1.213 | Pfmc-2TM Maurer's cleft two transmembrane protein | PFC0710w-a | inorganic pyrophosphatase, putative |
| MAL8P1.214 | stevor, pseudogene | PFC0715c | conserved Plasmodium protein, unknown function |
| MAL8P1.217 | stevor | PFC0831w | triosephophate isomerase, putative |
| MAL8P1.25 | conserved Plasmodium protein, unknown function | PFC0850c | endonuclease/exonuclease/phosphatase family protein, putative |
| MAL8P1.55 | conserved Plasmodium protein, unknown function | PFC0895w | CPW-WPC family protein |
| MAL8P1.59 | conserved Plasmodium protein, unknown function | PFC0905c | oocyst capsule protein |
| MAL8P1.6 | early transcribed membrane protein 8 | PFC0925w | conserved Plasmodium protein, unknown function |
| MAL8P1.61 | conserved Plasmodium protein, unknown function | PFC1080c | Pfmc-2TM Maurer's cleft two transmembrane protein |
| MAL8P1.75a | GTPase, putative | PFC1085c | Plasmodium exported protein (hyp4), unknown function |
| PF07_0003 | rifin | PFC1090w | Plasmodium exported protein (hyp5), unknown function |
| PF07_0006 | sporozoite threonine and asparagine-rich protein | PFC1105w | stevor (3D7-stevorT3-2) |
| PF07_0009 | chitinase precursor fragment, truncated | PFD0035c | stevor |
| PF07_0061 | conserved Plasmodium protein, unknown function | PFD0065w | stevor, pseudogene |
| PF07_0068 | cysteine desulfurase, putative | PFD0080c | Plasmodium exported protein (PHISTb), unknown function |
| PF07_0070 | drug metabolite transporter, putative | PFD0085c | acyl-CoA synthetase, PfACS6 |
| PF07_0081 | conserved Plasmodium protein, unknown function | PFD0120w | rifin, pseudogene |
| PF07_0100 | conserved Plasmodium protein, unknown function | PFD0135c | rifin, pseudogene |
| PF07_0103 | mitochondrial import inner membrane translocase subunit tim14, putative | PFD0155c | conserved Plasmodium protein, unknown function |
| PF07_0109 | conserved Plasmodium protein, unknown function | PFD0310w | sexual stage-specific protein precursor |
| PF07_0113 | conserved Plasmodium protein, unknown function | PFD0430c | perforin like protein 1 |
| PF07_0129 | acyl-coA synthetase, PfACS5 | PFD0440w | peptidase, M22 family, putative |
| PF07_0130 | stevor | PFD0465c | conserved Plasmodium protein, unknown function |
| PF08_0001 | Plasmodium exported protein, unknown function | PFD0480w | conserved Plasmodium protein, unknown function |
| PF08_0005 | conserved Plasmodium protein, unknown function | PFD0530c | GTPase, putative |
| PF08_0014 | apicoplast ribosomal protein L21 precursor, putative | PFD0555c | conserved Plasmodium protein, unknown function |
| PF08_0024 | conserved Plasmodium protein, unknown function | PFD0620c | rifin, pseudogene |
| PF08_0047 | conserved Plasmodium protein, unknown function | PFD0645w | rifin |
| PF08_0059 | protein kinase c inhibitor-like protein, putative | PFD0675w | apicoplast ribosomal protein L10 precursor, putative |
| PF08_0078 | ABC transporter, putative | PFD0690c | conserved Plasmodium membrane protein, unknown function |
| PF08_0116 | conserved Plasmodium protein, unknown function | PFD0710w | GTP binding protein, putative |
| PF10_0006 | rifin | PFD0760c | conserved Plasmodium protein, unknown function |
| PF10_0019 | early transcribed membrane protein 10.1 | PFD0780w | glutamyl-tRNA(Gln) amidotransferase subunit A, putative |
| PF10_0027 | conserved Plasmodium protein, unknown function | PFD0820w | conserved Plasmodium protein, unknown function |
| PF10_0035 | conserved Plasmodium protein, unknown function | PFD0930w | CGI-141 protein homolog, putative |
| PF10_0053 | methionine-tRNA ligase, putative | PFD0980w | holo-(acyl-carrier protein) synthase, putative |
| PF10_0055 | conserved Plasmodium protein, unknown function | PFD1037w | conserved Plasmodium protein, unknown function |
| PF10_0060 | conserved Plasmodium protein, unknown function | PFD1075w | serpentine receptor, putative |
| PF10_0070 | conserved Plasmodium membrane protein, unknown function | PFD1100c | conserved Plasmodium protein, unknown function |
| PF10_0082 | conserved Plasmodium membrane protein, unknown function | PFD1120c | early transcribed membrane protein 4 |
| PF10_0127 | conserved Plasmodium protein, unknown function | PFD1140w | Plasmodium exported protein (PHISTc), unknown function |
| PF10_0130 | conserved Plasmodium protein, unknown function | PFD1205w | Plasmodium exported protein (hyp15), unknown function |
| PF10_0134 | conserved Plasmodium protein, unknown function | PFE0020c | rifin |
| PF10_0139 | male gamete fusion factor HAP2, putative | PFE0030c | stevor, pseudogene |
| PF10_0157 | conserved Plasmodium protein, unknown function | PFE0080c | rhoptry-associated protein 2 |
| PF10_0164 | early transcribed membrane protein 10.3 | PFE0125w | conserved Plasmodium protein, unknown function |
| PF10_0175 | tRNA pseudouridine synthase, putative | PFE0205w | ATP-dependent helicase, putative |
| PF10_0177b | conserved Plasmodium protein, unknown function | PFE0265c | conserved Plasmodium protein, unknown function |
| PF10_0191 | tRNA methyltransferase, putative | PFE0355c | subtilisin-like protease 3, putative |
| PF10_0204 | conserved Plasmodium protein, unknown function | PFE0365c | conserved Plasmodium protein, unknown function |
| PF10_0207 | conserved Plasmodium membrane protein, unknown function | PFE0405c | longevity-assurance (LAG1) domain protein, putative |
| PF10_0208 | endomembrane protein 70, putative | PFE0475w | asparagine-tRNA ligase, putative |
| PF10_0221 | GcpE protein | PFE0565w | conserved Plasmodium protein, unknown function |
| PF10_0233 | conserved Plasmodium protein, unknown function | PFE0575c | conserved Plasmodium protein, unknown function |
| PF10_0246 | conserved Plasmodium protein, unknown function | PFE0710w | conserved Plasmodium protein, unknown function |
| PF10_0295 | conserved Plasmodium protein, unknown function | PFE0715w | aspartyl-tRNA synthetase, putative |
| PF10_0313 | mitochondrial preribosomal assembly protein rimM precursor, putative | PFE0815w | tRNA pseudouridine synthase, putative |
| PF10_0313a | conserved Plasmodium protein, unknown function | PFE0830c | sporozoite surface antigen MB2 |
| PF10_0317 | DER1-like protein, putative | PFE0855c | conserved Plasmodium protein, unknown function |
| PF10_0323 | early transcribed membrane protein 10.2 | PFE0905w | RAP protein, putative |
| PF10_0329 | plasmepsin VII | PFE1000c | conserved Plasmodium protein, unknown function |
| PF10_0332 | apicoplast ribosomal protein L27 precursor, putative | PFE1045c | conserved Plasmodium protein, unknown function |
| PF10_0344 | glutamate-rich protein | PFE1075c | conserved Plasmodium protein, unknown function |
| PF10_0390 | Pfmc-2TM Maurer's cleft two transmembrane protein | PFE1125w | mitochondrial ribosomal protein L17 precursor, putative |
| PF10_0391 | Plasmodium exported protein (hyp4), unknown function | PFE1130w | conserved protein, unknown function |
| PF10_0392 | Plasmodium exported protein (hyp5), unknown function | PFE1135w | iron-sulfur assembly protein, putative |
| PF10_0395 | stevor | PFE1205c | conserved Plasmodium membrane protein, unknown function |
| PF10_0397 | rifin | PFE1280w | conserved Plasmodium protein, unknown function |
| PF10_0401 | rifin | PFE1330c | conserved Plasmodium protein, unknown function |
| PF10_0403 | rifin | PFE1450c | conserved Plasmodium protein, unknown function |
| PF11_0013 | stevor, pseudogene | PFE1525w | conserved Plasmodium membrane protein, unknown function |
| PF11_0014 | Plasmodium falciparum Maurer's Cleft 2 transmembrane domain protein 11.1, PfMC-2TM_11.1 | PFE1590w | early transcribed membrane protein 5 |
| PF11_0023 | Plasmodium exported protein (hyp5), unknown function | PFE1615c | Plasmodium exported protein, unknown function |
| PF11_0024 | Plasmodium exported protein (hyp4), unknown function | PFE1620c | erythrocyte membrane protein 1, PfEMP1 |
| PF11_0025 | Pfmc-2TM Maurer's cleft two transmembrane protein | PFF0030c | erythrocyte membrane protein 1 (PfEMP1), pseudogene |
| PF11_0039 | early transcribed membrane protein 11.1 | PFF0050c | Plasmodium exported protein (hyp5), unknown function |
| PF11_0044 | iron-sulfur assembly protein, sufD, putative | PFF0055w | Plasmodium exported protein (hyp4), unknown function |
| PF11_0055 | conserved protein, unknown function | PFF0060w | Pfmc-2TM Maurer's cleft two transmembrane protein |
| PF11_0072 | apicoplast ribosomal protein S15 precursor, putative | PFF0110w | conserved Plasmodium protein, unknown function |
| PF11_0073 | conserved Plasmodium protein, unknown function | PFF0115c | elongation factor G, putative |
| PF11_0074 | exonuclease, putative | PFF0215w | conserved Plasmodium protein, unknown function |
| PF11_0076 | conserved Plasmodium protein, unknown function | PFF0230c | glyoxalase I |
| PF11_0157 | glycerol-3-phosphate dehydrogenase, putative | PFF0400w | conserved Plasmodium protein, unknown function |
| PF11_0167 | conserved Plasmodium membrane protein, unknown function | PFF0545c | conserved Plasmodium protein, unknown function |
| PF11_0174 | cathepsin C, homolog,dipeptidyl peptidase 1 | PFF0600w | conserved Plasmodium protein, unknown function |
| PF11_0175 | heat shock protein 101 | PFF0635w | conserved Plasmodium protein, unknown function |
| PF11_0181 | tyrosine-tRNA ligase, putative | PFF0650w | apicoplast ribosomal protein L18 precursor, putative |
| PF11_0197 | ankyrin repeat domain protein, putative | PFF0795w | conserved Plasmodium protein, unknown function |
| PF11_0209 | conserved Plasmodium protein, unknown function | PFF0850c | stevor |
| PF11_0212 | tRNA nucleotidyltransferase, putative | PFF0855c | rifin |
| PF11_0229 | conserved Plasmodium protein, unknown function | PFF1015w | conserved Plasmodium protein, unknown function |
| PF11_0238 | conserved Plasmodium protein, unknown function | PFF1070c | radical SAM protein, putative |
| PF11_0246 | conserved Plasmodium protein, unknown function | PFF1090c | conserved Plasmodium membrane protein, unknown function |
| PF11_0261 | conserved Plasmodium protein, unknown function | PFF1175c | conserved Plasmodium protein, unknown function |
| PF11_0270 | threonine -- tRNA ligase, putative | PFF1190c | N-acetylglucosaminylphosphatidylinositol deacetylase, putative |
| PF11_0285 | conserved Plasmodium protein, unknown function | PFF1195c | conserved Plasmodium protein, unknown function |
| PF11_0328 | conserved Plasmodium protein, unknown function | PFF1230c | conserved Plasmodium protein, unknown function |
| PF11_0348 | RNA (uracil-5-)methyltransferase, putative | PFF1395c | glutamyl-tRNA(Gln) amidotransferase subunit B, putative |
| PF11_0352 | protein disulfide isomerase | PFF1415c | DnaJ protein, putative |
| PF11_0361-b | conserved Plasmodium protein, unknown function | PFF1420w | phosphatidylcholine-sterol acyltransferase precursor, putative |
| PF11_0361-c | conserved Plasmodium protein, unknown function | PFF1475c | conserved Plasmodium protein, unknown function |
| PF11_0364 | conserved Plasmodium protein, unknown function | PFF1525c | Pfmc-2TM Maurer's cleft two transmembrane protein |
| PF11_0381 | subtilisin-like protease 2 | PFF1530c | Plasmodium exported protein (hyp4), unknown function |
| PF11_0386 | apicoplast ribosomal protein S14p/S29e precursor, putative | PFF1535w | Plasmodium exported protein (hyp5), unknown function |
| PF11_0411 | carbonic anhydrase, putative | PFF1550w | stevor |
| PF11_0441a | alpha/beta hydrolase, putative | PFF1560c | rifin |
| PF11_0459 | apicoplast import protein Tic20, putative | PFI0025c | rifin |
| PF11_0466 | transporter, putative | PFI0040c | erythrocyte membrane protein 1 (PfEMP1), exon2, pseudogene |
| PF11_0472 | conserved Plasmodium protein, unknown function | PFI0045c | stevor |
| PF11_0511 | Plasmodium exported protein, unknown function | PFI0070w | rifin |
| PF11_0516 | stevor | PFI0080w | stevor |
| PF11_0535 | conserved Plasmodium protein, unknown function | PFI0085c | Plasmodium exported protein (hyp5), unknown function |
| PF13_0012 | early transcribed membrane protein 13 | PFI0095c | serine/threonine protein kinase, FIKK family |
| PF13_0025 | conserved Plasmodium protein, unknown function | PFI0125c-a | serine/threonine protein kinase, FIKK family |
| PF13_0032 | conserved Plasmodium protein, unknown function | PFI0210c | conserved protein, unknown function |
| PF13_0040 | DNA-directed RNA polymerase alpha chain, putative | PFI0305c | conserved Plasmodium protein, unknown function |
| PF13_0073 | Plasmodium exported protein (hyp12), unknown function | PFI0375w | apicoplast ribosomal protein L35 precursor, putative |
| PF13_0077 | DEAD box helicase, putative | PFI0390c | conserved Plasmodium membrane protein, unknown function |
| PF13_0090 | ADP-ribosylation factor, putative | PFI0500w | conserved Plasmodium protein, unknown function |
| PF13_0100 | apicoplast glycerol-3-phosphate acyltransferase, putative | PFI0525w | nucleotide binding protein, putative |
| PF13_0125 | conserved Plasmodium protein, unknown function | PFI0565w | conserved Plasmodium protein, unknown function |
| PF13_0128 | beta-hydroxyacyl-ACP dehydratase precursor | PFI0570w | GTP binding protein, putative |
| PF13_0134 | conserved Plasmodium protein, unknown function | PFI0605c | conserved Plasmodium protein, unknown function |
| PF13_0141 | L-lactate dehydrogenase | PFI0640c | conserved Plasmodium protein, unknown function |
| PF13_0176 | apurinic/apyrimidinic endonuclease Apn1, putative | PFI0660c | protease, putative |
| PF13_0197a | reticulocyte binding protein homologue 6, pseudogene | PFI0670w | conserved Plasmodium protein, unknown function |
| PF13_0218a | conserved Plasmodium protein, unknown function | PFI0680c | arginyl-tRNA synthetase, putative |
| PF13_0223 | conserved protein, unknown function | PFI0685w | pseudouridylate synthase, putative |
| PF13_0272 | thioredoxin-related protein, putative | PFI0695c | phospholipid or glycerol acyltransferase, putative |
| PF13_0277 | conserved Plasmodium protein, unknown function | PFI0795w | conserved Plasmodium protein, unknown function |
| PF13_0312 | rhomboid protease ROM7 | PFI0840w | conserved Plasmodium membrane protein, unknown function |
| PF13_0353 | NADH-cytochrome b5 reductase, putative | PFI0890c-a | organelle ribosomal protein L3 precursor, putative |
| PF13_0354 | alanine--tRNA ligase, putative | PFI0890c-b | organelle ribosomal protein L3 precursor, putative |
| PF13_0361 | conserved Plasmodium protein, unknown function | PFI0900w | conserved Plasmodium protein, unknown function |
| PF14_0002 | rifin | PFI0920c | dihydrouridine synthase, putative |
| PF14_0005 | rifin | PFI0935w | DnaJ protein, putative |
| PF14_0007 | stevor | PFI0960w | dolichyl-diphosphooligosaccharide-protein glycosyltransferase, putative |
| PF14_0014 | Plasmodium exported protein, unknown function | PFI0990c | CS-domain containing protein, conserved in Apicomplexa |
| PF14_0016 | early transcribed membrane protein 14.1 | PFI1010w | conserved Plasmodium protein, unknown function |
| PF14_0031b | conserved Plasmodium protein, unknown function | PFI1050c | Fe-S-cluster redox enzyme, putative |
| PF14_0040 | secreted ookinete adhesive protein | PFI1095w | conserved Plasmodium protein, unknown function |
| PF14_0051 | DNA mismatch repair protein, putative | PFI1140w | flavodoxin-like protein |
| PF14_0061 | PPR repeat protein | PFI1205c | conserved Plasmodium membrane protein, unknown function |
| PF14_0088 | aldo-keto reductase, putative | PFI1240c | prolyl-t-RNA synthase, putative |
| PF14_0105 | conserved Plasmodium protein, unknown function | PFI1295c | monocarboxylate transporter, putative |
| PF14_0116 | conserved Plasmodium protein, unknown function | PFI1463w | conserved Plasmodium protein, unknown function |
| PF14_0133 | SufC ATPase, putative | PFI1468c | conserved Plasmodium protein, unknown function |
| PF14_0158 | conserved Plasmodium protein, unknown function | PFI1515w | selenoprotein |
| PF14_0166 | lysine-tRNA ligase, putative | PFI1530c | conserved Plasmodium protein, unknown function |
| PF14_0179 | liver specific protein 1, putative | PFI1580c | DHHC-type zinc finger protein, putative |
| PF14_0186 | conserved Plasmodium protein, unknown function | PFI1615w | conserved Plasmodium protein, unknown function |
| PF14_0198 | glycine-tRNA ligase, putative | PFI1645c | histidyl-tRNA synthetase, putative |
| PF14_0249 | conserved Plasmodium protein, unknown function | PFI1745c | early transcribed membrane protein |
| PF14_0250 | lipase, putative | PFI1765c | Plasmodium exported protein, unknown function |
| PF14_0265 | peptide chain release factor 1, putative | PFI1795c | Plasmodium exported protein, unknown function |
| PF14_0270 | apicoplast ribosomal protein L15 precursor, putative | PFL0035c | acyl-CoA synthetase, PfACS7 |
| PF14_0275 | conserved Plasmodium protein, unknown function | PFL0040c | serine/threonine protein kinase, FIKK family |
| PF14_0281 | plasmepsin IX | PFL0070c | Plasmodium exported protein, unknown function |
| PF14_0286 | glutamate dehydrogenase, putative | PFL0080c | NIMA related kinase 3 |
| PF14_0293 | conserved Plasmodium protein, unknown function | PFL0375w | conserved Plasmodium protein, unknown function |
| PF14_0318 | conserved Plasmodium protein, unknown function | PFL0380c | tRNA delta(2)-isopentenylpyrophosphate transferase, putative |
| PF14_0342 | conserved Plasmodium membrane protein, unknown function | PFL0400w | apicoplast ribosomal protein L29 precursor, putative |
| PF14_0348 | ATP-dependent Clp protease proteolytic subunit, putative | PFL0410w | cysteine repeat modular protein 3 |
| PF14_0351 | conserved Plasmodium protein, unknown function | PFL0500w | mitochondrial ribosomal protein L1-2 precursor, putative |
| PF14_0369 | copper transporter putative | PFL0595c | glutathione peroxidase |
| PF14_0415 | dephospho-CoA kinase, putative | PFL0600w | conserved Plasmodium protein, unknown function |
| PF14_0417 | heat shock protein 90, putative | PFL0655w | conserved Plasmodium membrane protein, unknown function |
| PF14_0421 | apicoplast 1-acyl-sn-glycerol-3-phosphate acyltransferase, putative | PFL0770w | seryl-tRNA synthetase, putative |
| PF14_0462 | ubiquitin-protein ligase, putative | PFL0795c | male development gene 1 |
| PF14_0495 | rhoptry neck protein 2 | PFL0805w | perforin like protein 2 |
| PF14_0506a | conserved protein, unknown function | PFL0945w | erythrocyte membrane protein 1 (PfEMP1), exon2, pseudogene |
| PF14_0554 | conserved Plasmodium protein, unknown function | PFL0973c | conserved Plasmodium protein, unknown function |
| PF14_0572 | conserved Plasmodium membrane protein, unknown function | PFL0990w | conserved Plasmodium protein, unknown function |
| PF14_0581-a | apicoplast ribosomal protein S10 precursor, putative | PFL1015w-a | conserved Plasmodium protein, unknown function |
| PF14_0581-b | apicoplast ribosomal protein S10 precursor, putative | PFL1015w-b | conserved Plasmodium protein, unknown function |
| PF14_0604a | conserved Plasmodium protein, unknown function | PFL1045w | conserved protein, unknown function |
| PF14_0625 | plasmepsin VIII | PFL1050w | conserved Plasmodium protein, unknown function |
| PF14_0629 | conserved Plasmodium protein, unknown function | PFL1060c | conserved protein, unknown function |
| PF14_0638 | conserved Plasmodium membrane protein, unknown function | PFL1210w | isoleucyl-tRNA synthetase, putative |
| PF14_0642 | organelle ribosomal protein L22/L17 precursor, putative | PFL1300c | conserved Plasmodium protein, unknown function |
| PF14_0658 | translation initiation factor EF-1, putative | PFL1390w | conserved Plasmodium protein, unknown function |
| PF14_0660 | protein phosphatase, putative | PFL1430c | conserved Plasmodium protein, unknown function |
| PF14_0675 | conserved Plasmodium protein, unknown function | PFL1540c | phenylalanyl-tRNA synthetase alpha chain, putative |
| PF14_0678 | exported protein 2 | PFL1660c | conserved Plasmodium protein, unknown function |
| PF14_0695 | DNA-directed RNA polymerase, alpha subunit, putative | PFL1670c | conserved Plasmodium protein, unknown function |
| PF14_0696 | conserved Plasmodium protein, unknown function | PFL1760w | conserved Plasmodium protein, unknown function |
| PF14_0729 | early transcribed membrane protein 14.2 | PFL1775c | s-adenosyl-methyltransferase, putative |
| PF14_0736 | Plasmodium exported protein, unknown function | PFL1780w | protein-S-isoprenylcysteine O-methyltransferase, putative |
| PF14_0742 | Plasmodium exported protein (hyp6), unknown function | PFL1945c | early transcribed membrane protein 12 |
| PF14_0751 | acyl-CoA synthetase | PFL1965w | product unspecified |
| PF14_0753 | Plasmodium exported protein (hyp13), unknown function | PFL2020c | conserved Plasmodium protein, unknown function |
| PF14_0757 | Plasmodium exported protein (PHISTa), unknown function | PFL2030w | queuine tRNA-ribosyltransferase, putative |
| PF14_0761 | acyl-CoA synthetase | PFL2115c | glucose inhibited division protein a homologue, putative |
| PF14_0767 | stevor | PFL2130w | conserved Plasmodium protein, unknown function |
| PF14_0771 | stevor | PFL2135c | conserved Plasmodium protein, unknown function |
| PF14_0791 | dfg10 like protein, putative | PFL2195w | clathrin coat assembly protein AP180, putative |
| PFA0055c | Plasmodium exported protein (hyp5), unknown function | PFL2205w | conserved Plasmodium protein, unknown function |
| PFA0060w | Plasmodium exported protein (hyp4), unknown function | PFL2290w | dipeptidyl peptidase 2, putative |
| PFA0065w | Pfmc-2TM Maurer's cleft two transmembrane protein | PFL2395c | apicoplast dimethyladenosine synthase, putative |
| PFA0090c | stevor | PFL2485c | tryptophanyl-tRNA synthetase, putative |
| PFA0115w | Plasmodium exported protein, unknown function | PFL2505c | conserved Plasmodium protein, unknown function |
| PFA0180w | ATP-dependent RNA helicase, putative | PFL2510w | chitinase |
| PFA0195w | parasite-infected erythrocyte surface protein | PFL2520w | reticulocyte binding protein homologue 3, pseudogene |
| PFA0200w | thrombospondin-related sporozoite protein | PFL2560c | Plasmodium exported protein, unknown function |
| PFA0210c | conserved Plasmodium protein, unknown function | PFL2565w | Plasmodium exported protein (PHISTa), unknown function |
| PFA0265c | conserved Plasmodium protein, unknown function | PFL2570w | acyl-CoA synthetase, PfACS3 |
| PFA0325w | conserved Plasmodium protein, unknown function | PFL2610w | stevor |
| PFA0340w | 2-C-methyl-D-erythritol 4-phosphate cytidylyltransferase, putative | PFL2620w | stevor |
| PFA0385w | conserved Plasmodium membrane protein, unknown function | PFL2630w | rifin |
| PFA0430c | secreted ookinete protein, putative | PFL2635w | stevor |
