# Supplementary material for: ApicoAP: The First Computational Model for Identifying Apicoplast-Targeted Proteins in Multiple Species of Apicomplexa
Source: PLoS One. 2012 May 4;7(5):e36598. doi: 10.1371/journal.pone.0036598 (PMC3344922; doi:10.1371/journal.pone.0036598)
Supplement: Table S10 — List of putative ApicoTPs for P. yoelii. (DOC) [file pone.0036598.s010.doc]

***Table S10: List of putative ApicoTPs for*** P. yoelii.

| **Gene id** | **EuPathDB product description** | **Gene id** | **EuPathDB product description** |
| --- | --- | --- | --- |
| PY00033 | DNA-directed RNA polymerase alpha chain | PY03792 | hypothetical protein |
| PY00041 | ribosomal protein L17, putative | PY03841 | Unknown protein |
| PY00133 | hypothetical protein | PY03856 | transmission-blocking target antigen s230 precursor |
| PY00138 | hypothetical protein | PY03869 | hypothetical protein |
| PY00161 | GTP-binding protein-related | PY03870 | hypothetical protein |
| PY00162 | MIF4G domain, putative | PY03959 | hypothetical protein |
| PY00185 | hypothetical protein | PY03989 | DHHC zinc finger domain, putative |
| PY00205 | early transcribed membrane protein family | PY04029 | hypothetical protein |
| PY00222 | CCAAT-box DNA binding protein subunit B | PY04089 | hypothetical protein |
| PY00228 | hypothetical protein | PY04145 | Peptidyl-tRNA hydrolase domain, putative |
| PY00235 | hypothetical protein | PY04164 | tRNA delta(2)-isopentenylpyrophosphate transferase, putative |
| PY00237 | hypothetical protein | PY04181 | hypothetical protein |
| PY00281 | hypothetical protein | PY04210 | hypothetical protein |
| PY00363 | glutamyl-tRNA synthetase | PY04246 | hypothetical protein |
| PY00434 | hypothetical protein | PY04254 | tyrosyl-tRNA synthetase 1-related |
| PY00465 | ribosomal protein L27, putative | PY04278 | hypothetical protein |
| PY00480 | signal peptidase 18 subunit-related | PY04280 | hypothetical protein |
| PY00557 | Clp protease | PY04294 | hypothetical protein |
| PY00597 | hypothetical protein | PY04295 | DNA-directed RNA polymerase |
| PY00607 | probable tRNA-methyltransferase | PY04317 | hypothetical protein |
| PY00619 | hypothetical protein | PY04333 | hypothetical protein |
| PY00688 | Ribosomal protein L15 amino terminal region, putative | PY04355 | hypothetical protein |
| PY00721 | Rab6 | PY04363 | probable tRNA modification GTPase trme |
| PY00739 | CCAAT-box DNA binding protein subunit B | PY04365 | hypothetical protein |
| PY00756 | triosephosphate isomerase, putative | PY04401 | hypothetical protein |
| PY00786 | hypothetical protein | PY04442 | hypothetical protein |
| PY00789 | NAD-dependent glycerol-3-phosphate dehydrogenase | PY04445 | malaria antigen-related |
| PY00802 | methionine aminopeptidase-like protein-related | PY04477 | hypothetical protein |
| PY00839 | Krueppel-like protein | PY04491 | hypothetical protein |
| PY00876 | hypothetical protein | PY04492 | hypothetical protein |
| PY00888 | hypothetical protein | PY04495 | 1st euk. member |
| PY00891 | tRNA nucleotidyltransferase, mitochondrial precursor | PY04602 | hypothetical protein |
| PY00895 | hypothetical protein | PY04640 | hypothetical protein |
| PY00905 | hypothetical protein | PY04658 | hypothetical protein |
| PY00907 | Uncharacterized protein family UPF0051, putative | PY04669 | hypothetical protein |
| PY00927 | prolyl-tRNA synthetase-related | PY04674 | similar to proteasome 26S subunit, non-ATPase, 10-related |
| PY00940 | hypothetical protein | PY04693 | hypothetical protein |
| PY00948 | hypothetical protein | PY04769 | hypothetical protein |
| PY00970 | Endonuclease/Exonuclease/phosphatase family, putative | PY04778 | ribosome releasing factor-related |
| PY01073 | hypothetical protein | PY04799 | early transcribed membrane protein family |
| PY01128 | hypothetical protein | PY04823 | hypothetical protein |
| PY01176 | hypothetical protein | PY04830 | neurofilament protein H form H2 |
| PY01193 | hypothetical protein | PY04834 | hypothetical protein |
| PY01204 | glutathione reductase | PY04836 | hypothetical protein |
| PY01244 | hypothetical protein | PY04866 | hypothetical protein |
| PY01268 | pepsinogen A-related | PY04886 | hypothetical protein |
| PY01291 | Drosophila melanogaster GH28477p-related | PY04929 | hypothetical protein |
| PY01304 | putative yir3 protein | PY04971 | cdk-related protein kinase 6-related |
| PY01325 | hypothetical protein | PY04983 | hypothetical protein |
| PY01378 | KED | PY05000 | hypothetical protein |
| PY01435 | hypothetical protein | PY05057 | hypothetical protein |
| PY01442 | putative yir1 protein | PY05092 | hypothetical protein |
| PY01461 | O1 | PY05127 | hypothetical protein |
| PY01479 | hypothetical protein | PY05129 | hypothetical protein |
| PY01488 | queuine tRNA-ribosyltransferase | PY05161 | hypothetical protein |
| PY01516 | hypothetical protein | PY05172 | hypothetical protein |
| PY01544 | hypothetical protein | PY05236 | UBX domain, putative |
| PY01557 | m1-family aminopeptidase | PY05249 | hypothetical protein |
| PY01563 | hypothetical protein | PY05329 | hypothetical protein |
| PY01566 | hypothetical protein | PY05368 | GTP binding protein-related |
| PY01586 | beta-hydroxyacyl-ACP dehydratase precursor | PY05402 | putative HSP protein |
| PY01593 | hypothetical protein | PY05406 | hypothetical protein |
| PY01612 | hypothetical protein | PY05427 | hypothetical protein |
| PY01616 | hypothetical protein | PY05433 | hypothetical protein |
| PY01664 | GcpE | PY05439 | hypothetical protein |
| PY01671 | 50S ribosomal protein L18 | PY05503 | similar to elaC homolog 1 |
| PY01683 | 5'-3' exonuclease, N-terminal resolvase-like domain, putative | PY05541 | hypothetical protein |
| PY01695 | acetyl-CoA carboxylase 1 precursor-related | PY05542 | s-adenosyl-methyltransferase mraw-related |
| PY01741 | Drosophila melanogaster BcDNA.LD06837, putative | PY05566 | hypothetical protein |
| PY01750 | 60S ribosomal protein L27 homolog | PY05568 | hypothetical protein |
| PY01826 | 36I5.4 | PY05577 | hypothetical protein |
| PY01863 | hypothetical protein | PY05596 | ump-cmp kinase |
| PY01880 | hypothetical protein | PY05600 | hypothetical protein |
| PY01887 | hypothetical protein | PY05646 | Drosophila melanogaster CG15040 gene product |
| PY01906 | heat shock protein 83 | PY05658 | lysyl-tRNA synthetase |
| PY01909 | dephospho-CoA kinase, putative | PY05692 | hypothetical protein |
| PY01929 | hypothetical protein | PY05714 | hypothetical protein |
| PY01930 | hypothetical protein | PY05725 | AP endonuclease 1 |
| PY01938 | PDZ domain protein | PY05754 | ribosomal protein S23 |
| PY01996 | hypothetical protein | PY05800 | hypothetical protein |
| PY02093 | hypothetical protein | PY05833 | hypothetical protein |
| PY02118 | hypothetical protein | PY05892 | Exp-2 |
| PY02122 | hypothetical protein | PY06003 | hypothetical protein |
| PY02126 | hypothetical protein | PY06054 | transport protein |
| PY02202 | hypothetical protein | PY06063 | hypothetical protein |
| PY02244 | hypothetical protein | PY06138 | hypothetical protein |
| PY02266 | GTP-binding protein | PY06142 | synthetic antigen of P.falciparum |
| PY02327 | hypothetical protein | PY06146 | hypothetical protein |
| PY02392 | hypothetical protein | PY06175 | hypothetical protein |
| PY02399 | hypothetical protein | PY06201 | hypothetical protein |
| PY02435 | ribosomal protein S14p/S29e, putative | PY06218 | hypothetical protein |
| PY02463 | hypothetical protein | PY06264 | hypothetical protein |
| PY02482 | hypothetical protein | PY06285 | hypothetical protein |
| PY02510 | PfSec61 | PY06350 | hypothetical protein |
| PY02552 | asparagine-rich protein | PY06355 | hypothetical protein |
| PY02587 | hypothetical protein | PY06389 | hypothetical protein |
| PY02589 | hypothetical protein | PY06437 | hypothetical protein |
| PY02621 | hypothetical protein | PY06448 | hypothetical protein |
| PY02622 | hypothetical protein | PY06454 | hypothetical protein |
| PY02689 | putative yir1 protein | PY06462 | ABC transporter, putative |
| PY02812 | hypothetical protein | PY06476 | hypothetical protein |
| PY02858 | Glu-tRNAGln amidotransferase subunit A, putative | PY06488 | hypothetical protein |
| PY02865 | hypothetical protein | PY06504 | hypothetical protein |
| PY02884 | O1 | PY06513 | hypothetical protein |
| PY02888 | hypothetical protein | PY06562 | hypothetical protein |
| PY02892 | hypothetical protein | PY06601 | hypothetical protein |
| PY02899 | hypothetical protein | PY06627 | hypothetical protein |
| PY02935 | hypothetical protein | PY06633 | hypothetical protein |
| PY02948 | hypothetical protein | PY06676 | hypothetical protein |
| PY03009 | hypothetical protein | PY06751 | hypothetical protein |
| PY03079 | hypothetical protein | PY06801 | Uncharacterized protein family UPF0034, putative |
| PY03081 | alanyl-tRNA synthetase, putative | PY06841 | hypothetical protein |
| PY03103 | hypothetical protein | PY06854 | hypothetical protein |
| PY03104 | hypothetical protein | PY06936 | hypothetical protein |
| PY03200 | hypothetical protein | PY06965 | hypothetical protein |
| PY03215 | hypothetical protein | PY06969 | hypothetical protein |
| PY03220 | hypothetical protein | PY06976 | hypothetical protein |
| PY03222 | hypothetical protein | PY06977 | similar to unknown proteins |
| PY03365 | hypothetical protein | PY07001 | ribosomal protein L3, putative |
| PY03369 | hypothetical protein | PY07026 | hypothetical protein |
| PY03383 | hypothetical protein | PY07040 | hypothetical protein |
| PY03403 | putative yir4 protein | PY07152 | hypothetical protein |
| PY03413 | hypothetical protein | PY07173 | hypothetical protein |
| PY03421 | Arabidopsis thaliana P42251 | PY07256 | hypothetical protein |
| PY03426 | Elongation factor Tu family, putative | PY07278 | hypothetical protein |
| PY03427 | hypothetical protein | PY07311 | hypothetical protein |
| PY03485 | L1P family of ribosomal proteins, putative | PY07338 | hypothetical protein |
| PY03498 | clustered-asparagine-rich protein | PY07368 | circumsporozoite protein precursor-related |
| PY03517 | hypothetical protein | PY07384 | Initiation factor 2 subunit family, putative |
| PY03547 | glutamyl-tRNA amidotransferase subunit b | PY07445 | translation elongation factor Ts |
| PY03555 | hypothetical protein | PY07452 | hypothetical protein |
| PY03582 | hypothetical protein | PY07461 | hypothetical protein |
| PY03622 | ribosomal protein S6, putative | PY07464 | hypothetical protein |
| PY03634 | hypothetical protein | PY07479 | hypothetical protein |
| PY03649 | hypothetical protein | PY07506 | Drosophila melanogaster CG12781 gene product |
| PY03652 | hypothetical protein | PY07509 | tRNA synthetases class I, putative |
| PY03655 | hypothetical protein | PY07551 | hypothetical protein |
| PY03657 | hypothetical protein | PY07607 | hypothetical protein |
| PY03715 | hypothetical protein | PY07621 | hypothetical protein |
| PY03733 | hypothetical protein | PY07673 | hypothetical protein |
| PY03763 | hypothetical protein | PY07828 | hypothetical protein |
| PY03767 | Helicase conserved C-terminal domain, putative | PY07837 | hypothetical protein |
| PY03768 | methionyl-tRNA formyltransferase homolog, putative |  |  |
