# Supplementary material for: ApicoAP: The First Computational Model for Identifying Apicoplast-Targeted Proteins in Multiple Species of Apicomplexa
Source: PLoS One. 2012 May 4;7(5):e36598. doi: 10.1371/journal.pone.0036598 (PMC3344922; doi:10.1371/journal.pone.0036598)
Supplement: Table S11 — List of putative ApicoTPs for B. bovis. (DOC) [file pone.0036598.s011.doc]

***Table S11: List of putative ApicoTPs for B. bovis***.

| **Gene id** | **EuPathDB product description** | **Gene id** | **EuPathDB product description** |
| --- | --- | --- | --- |
| BBOV_I000160 | conserved hypothetical protein | BBOV_III008210 | hypothetical protein |
| BBOV_I000200 | conserved hypothetical protein | BBOV_III008720 | membrane protein, putative |
| BBOV_I000310 | FAD binding domain containing protein | BBOV_III008780 | membrane protein, putative |
| BBOV_I000830 | conserved hypothetical protein | BBOV_III008960 | hypothetical protein |
| BBOV_I000990 | hypothetical protein | BBOV_III009160 | ER lumen protein retaining receptor |
| BBOV_I001010 | tRNA modification GTPase TrmE , putative | BBOV_III009440 | conserved hypothetical protein |
| BBOV_I001080 | hypothetical protein | BBOV_III009720 | hypothetical protein |
| BBOV_I001660 | hypothetical protein | BBOV_III010050 | ubiquitin family protein |
| BBOV_I001900 | conserved hypothetical protein | BBOV_III010320 | membrane protein, putative |
| BBOV_I002380 | hypothetical protein | BBOV_III010760 | hypothetical protein |
| BBOV_I002480 | hypothetical protein | BBOV_III010940 | peptidyl-prolyl cis-trans isomerase, cyclophilin-type f domain containing protein |
| BBOV_I002900 | hypothetical protein | BBOV_III011070 | hypothetical protein |
| BBOV_I003150 | hypothetical protein | BBOV_III011300 | hypothetical protein |
| BBOV_I003400 | hypothetical protein | BBOV_III011320 | hypothetical protein |
| BBOV_I003410 | ribosomal protein L21 domain containing protein | BBOV_III011330 | hypothetical protein |
| BBOV_I004140 | hypothetical protein | BBOV_III011340 | hypothetical protein |
| BBOV_I004180 | membrane protein, putative | BBOV_III011360 | hypothetical protein |
| BBOV_I004710 | membrane protein, putative | BBOV_III011580 | membrane protein, putative |
| BBOV_I004730 | ribosomal protein L3 domain containing protein | BBOV_III011590 | hypothetical protein |
| BBOV_I004860 | hypothetical protein | BBOV_III011750 | GTP-binding protein LepA family protein |
| BBOV_II000510 | transcription factor S-II protein, putative | BBOV_III011830 | membrane protein, putative |
| BBOV_II000720 | hypothetical protein | BBOV_III011870 | HIT domain containing protein |
| BBOV_II000770 | membrane protein, putative | BBOV_III011880 | membrane protein, putative |
| BBOV_II000970 | hypothetical protein | BBOV_IV000080 | membrane protein, putative |
| BBOV_II000990 | hypothetical protein | BBOV_IV000100 | membrane protein, putative |
| BBOV_II001040 | membrane protein, putative | BBOV_IV000420 | conserved hypothetical protein |
| BBOV_II001190 | hypothetical protein | BBOV_IV000480 | 50S ribosomal protein L33 |
| BBOV_II001200 | hypothetical protein | BBOV_IV001020 | DNA-directed RNA polymerase, alpha subunit, putative |
| BBOV_II001500 | membrane protein, putative | BBOV_IV001090 | hypothetical protein |
| BBOV_II002570 | membrane protein, putative | BBOV_IV001180 | hypothetical protein |
| BBOV_II002890 | p18 protein | BBOV_IV001340 | hypothetical protein |
| BBOV_II003910 | membrane protein, putative | BBOV_IV001660 | membrane protein, putative |
| BBOV_II003950 | CGI-141 protein-like protein, putative | BBOV_IV001670 | hypothetical protein |
| BBOV_II005150 | hypothetical protein | BBOV_IV001690 | aspartyl-tRNA synthetase, putative |
| BBOV_II005420 | hypothetical protein | BBOV_IV001950 | methionine aminopeptidase I, putative |
| BBOV_II005760 | tRNA synthetases class I protein superfamily | BBOV_IV002260 | membrane protein, putative |
| BBOV_II005780 | membrane protein, putative | BBOV_IV002270 | hypothetical protein |
| BBOV_II006010 | conserved unknown domain containing membrane protein | BBOV_IV002470 | membrane protein, putative |
| BBOV_II006390 | membrane protein, putative | BBOV_IV002870 | membrane protein, putative |
| BBOV_II006530 | membrane protein, putative | BBOV_IV002930 | adenylate kinase 2 |
| BBOV_II006670 | hypothetical protein | BBOV_IV003140 | conserved hypothetical protein |
| BBOV_II006950 | acyltransferase family protein | BBOV_IV003850 | ATP-dependent Clp protease proteolytic subunit, putative |
| BBOV_II007790 | hypothetical protein | BBOV_IV003890 | hypothetical protein |
| BBOV_III000030 | membrane protein, putative | BBOV_IV003930 | hypothetical protein |
| BBOV_III000390 | hypothetical protein | BBOV_IV003980 | hypothetical protein |
| BBOV_III000410 | predicted protein | BBOV_IV004070 | conserved hypothetical protein |
| BBOV_III000510 | patatin-like phospholipase family protein | BBOV_IV004090 | conserved membrane protein, putative |
| BBOV_III000530 | rhomboid family protein | BBOV_IV004170 | membrane protein, putative |
| BBOV_III000680 | membrane protein, putative | BBOV_IV004210 | membrane protein, putative |
| BBOV_III000740 | hypothetical protein | BBOV_IV004250 | membrane protein, putative |
| BBOV_III000830 | membrane protein, putative | BBOV_IV004270 | conserved, membrane protein, putative |
| BBOV_III000930 | FAD-dependent glycerol-3-phosphate dehydrogenase, putative | BBOV_IV004340 | membrane protein, putative |
| BBOV_III001180 | hypothetical protein | BBOV_IV004360 | membrane protein, putative |
| BBOV_III001240 | BBO225AA 22 kDa antigen, fragment | BBOV_IV004660 | hypothetical protein |
| BBOV_III001310 | membrane protein, putative | BBOV_IV004790 | membrane protein, putative |
| BBOV_III001500 | translation elongation factor Tu | BBOV_IV004890 | membrane protein, putative |
| BBOV_III002140 | membrane protein, putative | BBOV_IV005000 | cyclophilin, putative |
| BBOV_III002420 | DHHC zinc finger domain containing protein | BBOV_IV005200 | glycerol-3-phosphate-acyltransferase, putative |
| BBOV_III002560 | hypothetical protein | BBOV_IV005270 | membrane protein, putative |
| BBOV_III002660 | hypothetical protein | BBOV_IV005440 | membrane protein, putative |
| BBOV_III002870 | signal peptidase family protein | BBOV_IV005490 | conserved hypothetical protein |
| BBOV_III003260 | conserved hypothetical protein | BBOV_IV005750 | membrane protein, putative |
| BBOV_III003270 | biotin-requiring enzyme family protein | BBOV_IV005880 | ribosomal protein L35, putative |
| BBOV_III003400 | hypothetical protein | BBOV_IV005980 | hypothetical protein |
| BBOV_III003510 | eukaryotic aspartyl protease family protein | BBOV_IV006260 | GCC2 and GCC3 domain containing protein |
| BBOV_III003710 | hypothetical protein | BBOV_IV006500 | hypothetical protein |
| BBOV_III003920 | 50S ribosomal protein L15, putative | BBOV_IV007380 | conserved hypothetical protein |
| BBOV_III003930 | 50S ribosomal subunit protein L17, putative | BBOV_IV007480 | membrane protein, putative |
| BBOV_III004060 | hypothetical protein | BBOV_IV007620 | hypothetical protein |
| BBOV_III004210 | membrane protein, putative | BBOV_IV007780 | hypothetical protein |
| BBOV_III004280 | membrane protein, putative | BBOV_IV007840 | hypothetical protein |
| BBOV_III004330 | hypothetical protein | BBOV_IV007890 | aspartyl protease, putative |
| BBOV_III004470 | hypothetical protein | BBOV_IV008140 | ABC transporter ATP-binding protein |
| BBOV_III004490 | hypothetical protein | BBOV_IV008150 | 12D3 antigen |
| BBOV_III004510 | hypothetical protein | BBOV_IV008250 | conserved hypothetical protein |
| BBOV_III004950 | hypothetical protein | BBOV_IV008430 | hypothetical protein |
| BBOV_III005030 | membrane protein, putative | BBOV_IV008550 | p-type ATPase |
| BBOV_III005180 | hypothetical protein | BBOV_IV008770 | hypothetical protein |
| BBOV_III005360 | membrane protein, putative | BBOV_IV009020 | hypothetical protein |
| BBOV_III005620 | hypothetical protein | BBOV_IV009240 | hypothetical protein |
| BBOV_III005800 | hypothetical protein | BBOV_IV009580 | dephospho-CoA kinase, putative |
| BBOV_III005920 | membrane protein, putative | BBOV_IV009800 | ribosomal protein L11, putative |
| BBOV_III006200 | hypothetical protein | BBOV_IV010010 | hypothetical protein |
| BBOV_III006470 | hypothetical protein | BBOV_IV010210 | membrane protein, putative |
| BBOV_III006490 | hypothetical protein | BBOV_IV010360 | aspartyl protease, putative |
| BBOV_III006510 | hypothetical protein | BBOV_IV010380 | membrane protein, putative |
| BBOV_III006530 | hypothetical protein | BBOV_IV010740 | hypothetical protein |
| BBOV_III006630 | chaperonin, 10 kDa family protein | BBOV_IV010950 | tyrosyl-tRNA synthetase, putative |
| BBOV_III006710 | membrane protein, putative | BBOV_IV011030 | hypothetical protein |
| BBOV_III006770 | membrane protein, putative | BBOV_IV011540 | transmembrane CLPTM1 family protein |
| BBOV_III007060 | membrane protein, putative | BBOV_IV011680 | membrane protein, putative |
| BBOV_III007330 | hypothetical protein | BBOV_IV011780 | hypothetical protein |
| BBOV_III007380 | heat shock protein 90 | BBOV_IV011840 | membrane protein, putative |
| BBOV_III007420 | membrane protein, putative | BBOV_IV011890 | WD repeat domain containing protein |
| BBOV_III007430 | 30S ribosomal protein S15 | BBOV_IV012010 | conserved hypothetical protein |
| BBOV_III007790 | casein kinase II, alpha chain (CK II) | BBOV_IV012080 | tRNA pseudouridine synthase, putative |
| BBOV_III008010 | conserved hypothetical protein | BBOV_IV012110 | hypothetical protein |
