# Supplementary material for: ApicoAP: The First Computational Model for Identifying Apicoplast-Targeted Proteins in Multiple Species of Apicomplexa
Source: PLoS One. 2012 May 4;7(5):e36598. doi: 10.1371/journal.pone.0036598 (PMC3344922; doi:10.1371/journal.pone.0036598)
Supplement: Table S12 — List of putative ApicoTPs for T. gondii. (DOC) [file pone.0036598.s012.doc]

***Table S12: List of putative ApicoTPs for*** T. gondii.

| **Gene id** | **EuPathDB product description** | **Gene id** | **EuPathDB product description** |
| --- | --- | --- | --- |
| TGME49_000230 | PAN domain-containing protein | TGME49_054200 | glycyl-tRNA synthetase, putative |
| TGME49_000240 | PAN domain-containing protein | TGME49_054270 | hypothetical protein |
| TGME49_000270 | PAN domain-containing protein | TGME49_054540 | CDP-alcohol phosphatidyltransferase domain-containing protein |
| TGME49_000360 | hypothetical protein | TGME49_054620 | 60S ribosomal protein L39, putative |
| TGME49_000440 | hypothetical protein | TGME49_054670 | hypothetical protein |
| TGME49_000600 | hypothetical protein | TGME49_055160 | hypothetical protein |
| TGME49_001100 | hypothetical protein | TGME49_055360 | hypothetical protein |
| TGME49_001180 | megakaryocyte stimulating factor, putative | TGME49_055650 | hypothetical protein |
| TGME49_002050 | hypothetical protein | TGME49_055910 | hypothetical protein, conserved |
| TGME49_002290 | hypothetical protein, conserved | TGME49_056000 | endoplasmic reticulum retention receptor, putative |
| TGME49_002530 | aspartyl-tRNA synthetase, putative | TGME49_056980 | glycerol-3-phosphate acyltransferase, putative |
| TGME49_002570 | ribophorin I, putative | TGME49_057380 | hypothetical protein |
| TGME49_002620 | hypothetical protein | TGME49_058190 | hypothetical protein |
| TGME49_002800 | cytochrome c oxidase assembly protein COX11, putative | TGME49_058580 | Rhoptry kinase family protein ROP17 |
| TGME49_002860 | hypothetical protein, conserved | TGME49_058750 | hypothetical protein |
| TGME49_003600 | hypothetical protein | TGME49_058860 | hypothetical protein |
| TGME49_003720 | vitamin K epoxide reductase complex subunit 1, putative | TGME49_059030 | hypothetical protein |
| TGME49_003930 | hypothetical protein | TGME49_059040 | hypothetical protein |
| TGME49_004370 | hypothetical protein | TGME49_059620 | hypothetical protein |
| TGME49_004410 | endonuclease/exonuclease/phosphatase domain-containing protein | TGME49_059960 | GDA1/CD39 (nucleoside phosphatase) family domain containing protein |
| TGME49_004550 | hypothetical protein | TGME49_060290 | hypothetical protein |
| TGME49_005360 | hypothetical protein | TGME49_061040 | hypothetical protein |
| TGME49_005450 | hypothetical protein | TGME49_061420 | hypothetical protein, conserved |
| TGME49_005680 | transmembrane domain-containing protein | TGME49_061970 | hypothetical protein |
| TGME49_005760 | hypothetical protein, conserved | TGME49_062090 | hypothetical protein |
| TGME49_006350 | ER lumen protein retaining receptor 1, putative | TGME49_062560 | hypothetical protein |
| TGME49_006390 | hypothetical protein | TGME49_062930 | hypothetical protein |
| TGME49_006630 | hypothetical protein | TGME49_063120 | hypothetical protein |
| TGME49_007180 | indole-3-glycerol phosphate synthase domain containing protein | TGME49_063730 | glycerol-3-phosphate dehydrogenase, putative |
| TGME49_007740 | hypothetical protein | TGME49_063870 | glutamyl-tRNA synthetase, putative |
| TGME49_007900 | transcription factor IIIB subunit, putative | TGME49_064040 | hypothetical protein, conserved |
| TGME49_008330 | hypothetical protein | TGME49_064090 | hypothetical protein |
| TGME49_008370 | myosin heavy chain, putative | TGME49_064930 | hypothetical protein |
| TGME49_008560 | mitochondrial carrier domain-containing protein | TGME49_065520 | hypothetical protein |
| TGME49_008740 | microneme protein, putative | TGME49_066110 | ATP-dependent RNA helicase, putative |
| TGME49_008770 | hypothetical protein | TGME49_066750 | hypothetical protein, conserved |
| TGME49_009060 | thrombospondin type 1 domain-containing protein | TGME49_067130 | SRS38A |
| TGME49_009150 | mitochondrial alternative NADH dehydrogenase 1 | TGME49_067380 | UDP-N-acetylglucosamine transporter, putative |
| TGME49_009180 | PAN domain-containing protein, putative | TGME49_067410 | scavenger receptor protein TgSR1, putative |
| TGME49_009240 | hypothetical protein, conserved | TGME49_067790 | hypothetical protein |
| TGME49_009620 | eukaryotic aspartyl protease, putative | TGME49_068030 | hypothetical protein |
| TGME49_009720 | hypothetical protein | TGME49_068390 | hypothetical protein |
| TGME49_009810 | hypothetical protein | TGME49_068700 | hypothetical protein |
| TGME49_009980 | Rhoptry kinase family protein ROP42 (incomplete catalytic triad) | TGME49_068780 | hypothetical protein |
| TGME49_010090 | Rhoptry kinase family protein ROP43 (incomplete catalytic triad) | TGME49_068890 | citrate synthase, putative |
| TGME49_010110 | Rhoptry kinase family protein ROP44 (incomplete catalytic triad) | TGME49_069050 | hypothetical protein, conserved |
| TGME49_010370 | RNA helicase-1 | TGME49_069070 | hypothetical protein |
| TGME49_011450 | hypothetical protein, conserved | TGME49_069120 | oxidoreductase, putative |
| TGME49_011640 | hypothetical protein | TGME49_069150 | zinc finger DHHC domain-containing protein |
| TGME49_011710 | TB2/DP1, HVA22 domain-containing protein | TGME49_069380 | hypothetical protein |
| TGME49_012100 | molybdenum cofactor synthesis protein, putative | TGME49_069400 | oxidoreductase, short chain dehydrogenase/reductase domain-containing protein |
| TGME49_012160 | hypothetical protein | TGME49_069690 | hypothetical protein |
| TGME49_012200 | hypothetical protein | TGME49_069860 | hypothetical protein |
| TGME49_013010 | hypothetical protein | TGME49_069980 | protein transport protein Sec61 alpha subunit isoform 1, putative |
| TGME49_013050 | hypothetical protein | TGME49_070220 | hypothetical protein |
| TGME49_013090 | sec1 family domain-containing protein | TGME49_070510 | asparaginyl-tRNA synthetase, putative |
| TGME49_013260 | hypothetical protein | TGME49_070910 | glycerol-3-phosphate acyltransferase, putative |
| TGME49_013320 | hypothetical protein | TGME49_071070 | cysteine protease domain containing protein |
| TGME49_013340 | glucose-methanol-choline oxidoreductase domain-containing protein | TGME49_071150 | hypothetical protein |
| TGME49_014290 | intracellular protease, putative | TGME49_071480 | hypothetical protein |
| TGME49_014380 | hypothetical protein | TGME49_071610 | pyrroline-5-carboxylase reductase, putative |
| TGME49_014570 | hypothetical protein | TGME49_071620 | hypothetical protein |
| TGME49_014780 | bis(5'-nucleosyl)-tetraphosphatase (asymmetrical), putative | TGME49_071760 | thioredoxin domain-containing protein |
| TGME49_015490 | transporter, major facilitator family domain containing protein | TGME49_071860 | tRNA (Uracil-5-)-methyltransferase domain-containing protein |
| TGME49_015510 | S-adenosyl-methyltransferase mraW, putative | TGME49_072170 | hypothetical protein |
| TGME49_015520 | hypothetical protein | TGME49_072180 | hypothetical protein |
| TGME49_015540 | hypothetical protein, conserved | TGME49_072240 | hypothetical protein |
| TGME49_015690 | hypothetical protein | TGME49_073270 | hypothetical protein |
| TGME49_015910 | hypothetical protein | TGME49_073910 | hypothetical protein, conserved |
| TGME49_015940 | acetyl-CoA transporter, putative | TGME49_073930 | SWIB/MDM2 domain-containing protein |
| TGME49_016100 | hypothetical protein | TGME49_073980 | hypothetical protein |
| TGME49_016180 | hypothetical protein | TGME49_075410 | hypothetical protein, conserved |
| TGME49_016460 | hypothetical protein | TGME49_075700 | hypothetical protein |
| TGME49_016720 | hypothetical protein | TGME49_075710 | hypothetical protein |
| TGME49_016740 | hypothetical protein | TGME49_075730 | hypothetical protein |
| TGME49_017040 | hypothetical protein | TGME49_075770 | hypothetical protein |
| TGME49_017160 | hypothetical protein, conserved | TGME49_076910 | endoplasmic reticulum retention receptor, putative |
| TGME49_017520 | hypothetical protein | TGME49_077030 | isoleucyl-tRNA synthetase, putative |
| TGME49_017590 | hypothetical protein | TGME49_078150 | hypothetical protein |
| TGME49_018270 | hypothetical protein | TGME49_078460 | hypothetical protein |
| TGME49_018740 | hypothetical protein | TGME49_078510 | protein phosphatase 2C, putative |
| TGME49_018860 | hypothetical protein | TGME49_078620 | hypothetical protein |
| TGME49_020240 | proline-rich protein | TGME49_078680 | hypothetical protein |
| TGME49_020300 | ribosomal protein L15, putative | TGME49_078690 | hypothetical protein |
| TGME49_021350 | hypothetical protein | TGME49_080520 | hypothetical protein |
| TGME49_021680 | hypothetical protein | TGME49_080670 | hypothetical protein |
| TGME49_021710 | TBC domain-containing protein | TGME49_080720 | hypothetical protein |
| TGME49_022080 | hypothetical protein | TGME49_080730 | nucleotide-binding protein, putative |
| TGME49_022120 | hypothetical protein | TGME49_081350 | hypothetical protein, conserved |
| TGME49_022270 | hypothetical protein | TGME49_081370 | hypothetical protein, conserved |
| TGME49_022300 | hypothetical protein | TGME49_081590 | hypothetical protein |
| TGME49_022670 | hypothetical protein | TGME49_081600 | hypothetical protein |
| TGME49_022930 | hypothetical protein | TGME49_081780 | hypothetical protein |
| TGME49_022940 | oocyst wall protein, putative | TGME49_082170 | hypothetical protein |
| TGME49_023010 | hypothetical protein | TGME49_083450 | hypothetical protein |
| TGME49_023250 | vacuolar type H+-ATPase proteolipid subunit, putative | TGME49_083760 | hypothetical protein |
| TGME49_023600 | hypothetical protein | TGME49_084580 | ribose-phosphate pyrophosphokinase, putative |
| TGME49_023830 | fasciclin domain-containing protein | TGME49_084660 | hypothetical protein, conserved |
| TGME49_023850 | hypothetical protein | TGME49_084780 | hypothetical protein |
| TGME49_023920 | hypothetical protein | TGME49_085240 | trans-2,3-enoyl-CoA reductase, putative |
| TGME49_024080 | kazal-type serine protease inhibitor domain-containing protein | TGME49_085290 | hypothetical protein |
| TGME49_024820 | hypothetical protein | TGME49_085530 | ribosomal protein L35 domain-containing protein |
| TGME49_025140 | folate/methotrexate transporter FT1, putative | TGME49_086180 | methionine tRNA synthetase, putative |
| TGME49_025200 | trichohyalin, putative | TGME49_086610 | 30S ribosomal protein S14, putative |
| TGME49_025250 | ribosomal protein L14p/L2 domain containing protein | TGME49_086660 | kinesin heavy chain, putative |
| TGME49_025330 | hypothetical protein | TGME49_086740 | microneme protein, putative |
| TGME49_025550 | phosphatidylserine decarboxylase proenzyme, putative | TGME49_086770 | hypothetical protein, conserved |
| TGME49_025730 | hypothetical protein | TGME49_087180 | hypothetical protein |
| TGME49_025830 | hypothetical protein | TGME49_087250 | hypothetical protein |
| TGME49_026070 | hypothetical protein, conserved | TGME49_088000 | hypothetical protein |
| TGME49_026270 | hypothetical protein | TGME49_088680 | endonuclease V, putative |
| TGME49_026370 | dgat2l1-prov protein | TGME49_089130 | hypothetical protein |
| TGME49_026590 | hypothetical protein | TGME49_089870 | hypothetical protein |
| TGME49_026740 | zinc finger (C3HC4 RING finger) protein, putative | TGME49_090700 | hypothetical protein |
| TGME49_026790 | ABC transporter, putative | TGME49_092400 | hypothetical protein |
| TGME49_026920 | hypothetical protein | TGME49_092970 | hypothetical protein |
| TGME49_027020 | NAD-dependent deacetylase, putative | TGME49_093040 | hypothetical protein |
| TGME49_027310 | hypothetical protein | TGME49_093510 | poly(ADP)-ribose polymerase domain-containing protein |
| TGME49_027370 | X-Pro dipeptidyl-peptidase domain-containing protein | TGME49_094040 | hypothetical protein |
| TGME49_027410 | hypothetical protein | TGME49_094400 | hypothetical protein |
| TGME49_027640 | hypothetical protein, conserved | TGME49_095060 | hypothetical protein |
| TGME49_028050 | hypothetical protein | TGME49_095090 | hypothetical protein |
| TGME49_028110 | hypothetical protein | TGME49_095460 | got1-like family domain-containing protein |
| TGME49_028170 | serine/threonine protein phosphatase, putative | TGME49_095720 | sulfite oxidase, putative |
| TGME49_028350 | elongation factor Tu GTP binding domain-containing protein | TGME49_095770 | hypothetical protein |
| TGME49_028500 | high-affinity cGMP-specific 3',5'-cyclic phosphodiesterase 9A, putative | TGME49_095910 | ATP-dependent Clp protease adaptor domain-containing protein |
| TGME49_030100 | hypothetical protein | TGME49_097100 | hypothetical protein |
| TGME49_030180 | hypothetical protein | TGME49_097160 | hypothetical protein |
| TGME49_030590 | beta-1,4-mannosyltransferase, putative | TGME49_097280 | hypothetical protein, conserved |
| TGME49_030920 | clathrin coat assembly protein AP50, putative | TGME49_097510 | hypothetical protein |
| TGME49_031110 | hypothetical protein | TGME49_097650 | serine/threonine protein phosphatase, putative |
| TGME49_031130 | hypothetical protein | TGME49_097880 | hypothetical protein |
| TGME49_031220 | hypothetical protein | TGME49_099140 | hypothetical protein |
| TGME49_031360 | hypothetical protein | TGME49_099220 | hypothetical protein, conserved |
| TGME49_031450 | hypothetical protein | TGME49_099570 | hypothetical protein |
| TGME49_032110 | hypothetical protein | TGME49_100230 | hypothetical protein |
| TGME49_032170 | hypothetical protein | TGME49_100250 | mtN3/saliva family domain-containing protein |
| TGME49_032570 | hypothetical protein | TGME49_100320 | dimethyladenosine synthase, putative |
| TGME49_033430 | hypothetical protein | TGME49_101130 | hypothetical protein |
| TGME49_033460 | SRS29B (= SAG1, P30) | TGME49_101210 | NAD(P) transhydrogenase, alpha subunit, putative |
| TGME49_033750 | hypothetical protein | TGME49_101230 | DNA repair protein, putative |
| TGME49_033840 | hypothetical protein | TGME49_101370 | zinc finger DHHC domain-containing protein, conserved |
| TGME49_034060 | hypothetical protein | TGME49_101580 | hypothetical protein, conserved |
| TGME49_034210 | hypothetical protein | TGME49_101690 | hypothetical protein |
| TGME49_034330 | hypothetical protein, conserved | TGME49_102350 | hypothetical protein |
| TGME49_034340 | hypothetical protein, conserved | TGME49_104250 | peptidyl-prolyl cis-trans isomerase, putative |
| TGME49_035180 | hypothetical protein | TGME49_104450 | hypothetical protein |
| TGME49_035390 | PAN domain-containing protein | TGME49_104470 | oxidoreductase, putative |
| TGME49_035660 | hypothetical protein, conserved | TGME49_104920 | hypothetical protein, conserved |
| TGME49_036190 | hypothetical protein | TGME49_105020 | hypothetical protein |
| TGME49_036290 | hypothetical protein | TGME49_105120 | sodium:solute symporter family domain-containing protein |
| TGME49_037080 | hypothetical protein | TGME49_105130 | hypothetical protein |
| TGME49_037130 | cytochrome b, putative | TGME49_105540 | hypothetical protein |
| TGME49_038950 | fatty acyl-CoA desaturase, putative | TGME49_105870 | dolichyl-diphosphooligosaccharide--protein glycotransferase, putative |
| TGME49_039010 | hypothetical protein | TGME49_106520 | tRNA pseudouridine synthase B, putative |
| TGME49_039880 | hypothetical protein | TGME49_108020 | SRS57 (= SAG3, P43) |
| TGME49_040200 | hypothetical protein, conserved | TGME49_108950 | histidine acid phosphatase domain containing protein |
| TGME49_040390 | CAM kinase, CDPK family | TGME49_109110 | tRNA methyltransferase domain-containing protein |
| TGME49_040440 | hypothetical protein | TGME49_109580 | hypothetical protein, conserved |
| TGME49_040520 | hypothetical protein | TGME49_109760 | hypothetical protein |
| TGME49_040720 | hypothetical protein | TGME49_109900 | hypothetical protein |
| TGME49_040860 | 1-acyl-sn-glycerol-3-phosphate acyltransferase, putative | TGME49_109910 | hypothetical protein |
| TGME49_041140 | DEAD/DEAH box helicase, putative | TGME49_109990 | hypothetical protein, conserved |
| TGME49_041190 | hypothetical protein | TGME49_110140 | hypothetical protein |
| TGME49_041300 | hypothetical protein | TGME49_110350 | hypothetical protein |
| TGME49_041610 | hypothetical protein | TGME49_110390 | hypothetical protein |
| TGME49_041870 | glutamyl-tRNA synthetase, putative | TGME49_110870 | hypothetical protein, conserved |
| TGME49_042270 | NUDIX hydrolase domain-containing protein | TGME49_110990 | hypothetical protein |
| TGME49_042440 | hypothetical protein | TGME49_111170 | hypothetical protein |
| TGME49_042590 | hypothetical protein | TGME49_111510 | PIK3R4 kinase-related protein (incomplete catalytic triad) |
| TGME49_042820 | hypothetical protein | TGME49_111670 | hypothetical protein |
| TGME49_043110 | hypothetical protein | TGME49_111840 | hypothetical protein |
| TGME49_043480 | 50S ribosomal protein L3, putative | TGME49_112040 | hypothetical protein |
| TGME49_043760 | hypothetical protein, conserved | TGME49_112180 | hypothetical protein, conserved |
| TGME49_044050 | hypothetical protein | TGME49_112510 | hypothetical protein |
| TGME49_044140 | hypothetical protein | TGME49_112580 | hypothetical protein |
| TGME49_044630 | hypothetical protein | TGME49_112690 | hypothetical protein |
| TGME49_045510 | P-type ATPase, putative | TGME49_112700 | hypothetical protein |
| TGME49_045550 | hypothetical protein | TGME49_113050 | short-chain dehydrogenase/reductase family protein, putative |
| TGME49_045600 | hypothetical protein | TGME49_113540 | hypothetical protein |
| TGME49_046180 | hypothetical protein | TGME49_113630 | hypothetical protein |
| TGME49_046550 | eukaryotic aspartyl protease, putative | TGME49_113930 | hypothetical protein, conserved |
| TGME49_047000 | TPR domain-containing protein | TGME49_114020 | hypothetical protein |
| TGME49_047210 | hypothetical protein | TGME49_114330 | ABC transporter, putative |
| TGME49_047270 | hypothetical protein | TGME49_114670 | hypothetical protein |
| TGME49_047350 | thioredoxin, putative | TGME49_114850 | hypothetical protein, conserved |
| TGME49_047470 | nucleolar protein 5A, putative | TGME49_115310 | hypothetical protein |
| TGME49_047520 | hypothetical protein, conserved | TGME49_115360 | hypothetical protein |
| TGME49_047530 | hypothetical protein | TGME49_115730 | apical membrane antigen, putative |
| TGME49_047680 | hypothetical protein, conserved | TGME49_116550 | hypothetical protein |
| TGME49_048320 | mitochondrial carrier family protein | TGME49_116690 | hypothetical protein |
| TGME49_048380 | hypothetical protein | TGME49_118650 | transhydrogenase, putative |
| TGME49_048410 | hypothetical protein | TGME49_118680 | 3',5'--cyclic-nucleotide phosphodiesterase, putative |
| TGME49_048510 | hypothetical protein | TGME49_119340 | kelch motif domain-containing protein |
| TGME49_048900 | hypothetical protein | TGME49_119630 | hypothetical protein |
| TGME49_049160 | hypothetical protein | TGME49_119660 | hypothetical protein |
| TGME49_049760 | vitamin K epoxide reductase complex subunit 1, putative | TGME49_119680 | hypothetical protein |
| TGME49_050050 | hypothetical protein | TGME49_119740 | hypothetical protein |
| TGME49_050110 | hypothetical protein, conserved | TGME49_120150 | GTP-binding protein 1, putative |
| TGME49_051400 | transmembrane protein, putative | TGME49_120190 | SRS16B (= SRS9) |
| TGME49_051570 | CAAX amino terminal protease family domain-containing protein | TGME49_120440 | hypothetical protein |
| TGME49_051770 | hypothetical protein | TGME49_120530 | hypothetical protein |
| TGME49_051900 | hypothetical protein, conserved | TGME49_120540 | hypothetical protein |
| TGME49_052070 | hypothetical protein | TGME49_120730 | homoserine O-acetyltransferase, putative |
| TGME49_052190 | hypothetical protein, conserved | TGME49_120770 | hypothetical protein |
| TGME49_052360 | Rhoptry kinase family protein ROP24 (incomplete catalytic triad) | TGME49_121280 | hypothetical protein |
| TGME49_053330 | Rhoptry kinase family protein, truncated (incomplete catalytic triad) | TGME49_121310 | 16S rRNA processing protein RimM, putative |
| TGME49_053600 | hypothetical protein | TGME49_121700 | hypothetical protein |
| TGME49_053880 | GNS1/SUR4 family domain-containing protein |  |  |
